# Antibacterial Interventions for Orthodontic Appliances; Surface Modifications, Coatings and Bulk-Incorporated Antibacterial Agents: Materials, Mechanisms and Clinical Application—A Scoping Review

**DOI:** 10.3390/ma19173644

**Published:** 2026-08-27

**Authors:** Berta Furió-Alonso, Javier Gil, Danica Nikolic Jovanovic, Andreu Puigdollers-Pérez

**Affiliations:** 1 Department of Orthodontics, Faculty of Dentistry, Universitat Internacional de Catalunya, 08195 Barcelona, Spain; danicanikolic@uic.es (D.N.J.); andreup@uic.es (A.P.-P.); 2 Bioinspired Oral Biomaterials and Interfaces (BOBI), Department of Materials Science and Engineering, UPC, Universitat Politècnica de Catalunya-Barcelona Tech, 08019 Barcelona, Spain; javier.gil.mur@upc.edu

**Keywords:** antibacterial coatings, orthodontic appliances, silver nanoparticles, titanium dioxide, biofilm inhibition, white spot lesions, nanoparticles, scoping review, evidence mapping

## Abstract

**Highlights:**

- Across 96 in vitro and 13 in vivo studies, antibacterial coatings on orthodontic appliance surfaces consistently and significantly reduce bacterial growth and adhesion compared to uncoated controls, with most coatings achieving 50–99% reductions in colony counts.
- Durability varies substantially by coating type with elution-dependent systems losing efficacy over months while contact-active and bulk-distributed coatings maintain longer-term protection.
- Under the laboratory conditions evaluated, nitrogen-doped TiO_2_ demonstrated favorable combinations of antibacterial activity, durability, and reported biocompatibility. However, long-term clinical effectiveness has not been established.
- TiN-Cu and layered polydopamine/PEDOT/AgNP systems seem to balance high antibacterial efficacy, sustained activity over 4–6 weeks, and demonstrated biocompatibility, though all evidence remains preclinical, and these coatings have not yet been validated in clinical use.
- Combination coatings of silver and metal oxides (particularly Ag/ZnO and Ag/TiO_2_) and nitrogen-doped TiO_2_ showed a favorable balance of antibacterial activity and reported biocompatibility under controlled laboratory conditions; however, clinical validation remains lacking, while pure TiO_2_ coatings are limited by UV dependence and the rutile phase of TiO_2_ show higher levels of cytotoxicity, based on a single in vitro study; this phase-dependent difference is highly subject to particle size, dose, agglomeration state, and preparation conditions.

**Main findings**

- Antibacterial coatings, particularly silver-based, nitrogen-doped titanium dioxide, and copper oxide formulations, consistently and significantly reduce bacterial adhesion and biofilm formation on orthodontic device surfaces compared to uncoated controls in vitro, though clinical validation through human trials remains lacking.
- Antibacterial coatings reliably reduce bacterial adhesion and biofilm formation on orthodontic devices compared to uncoated controls, with silver nanoparticles bulk-incorporated into acrylic appliance bases or combined with structural matrices (such as Ag/ZnO, Ag+ TiO_2_, or CS-Ag composites) showing favorable laboratory profiles with greater durability among the coating types investigated, while surface-deposited photocatalytic coatings like TiO_2_ show strong in vitro results but frequently fail in vivo due to mechanical delamination and light-dependence, and all major coating types demonstrate acceptable toxicity at orthodontically relevant concentrations with the exception of TiO_2_ rutile phase (based on a single in vitro study; TiO_2_ cytotoxicity is highly dependent on particle size, dose, and preparation conditions) and high-concentration silver formulations lacking antifouling modifications.

**Implications of the main findings**

- Nitrogen-doped TiO_2_ and silver/zinc oxide composite coatings on metallic brackets demonstrated a favorable balance of antibacterial activity and reported biocompatibility under laboratory conditions; however, clinical validation remains lacking.
- Antibacterial coatings on orthodontic devices consistently reduce bacterial adhesion and biofilm formation in vitro compared to uncoated controls, but clinical in vivo evidence remains limited and shows more modest, time-dependent effects than laboratory data suggest.

**Abstract:**

Biofilm accumulation on orthodontic appliances is a recognized risk factor for white spot lesion formation and caries during treatment. Antibacterial surface modifications and coating strategies have been proposed as appliance-level preventive approaches. Studied interventions in the literature span true thin-film surface coatings, plasma-based and ion-implantation surface modifications, and bulk-incorporated antibacterial agents in appliance matrix materials. Yet the extent to which in vitro efficacy translates to clinically meaningful protection remains unresolved. This PRISMA-ScR-compliant scoping review searched PubMed/MEDLINE, Scopus, Web of Science, Cochrane Library, and Google Scholar, identifying 109 eligible studies: 96 in vitro, 8 in vivo animal studies and 5 clinical trials, covering brackets, archwires, clear aligners, bands, miniscrews, elastomeric ligatures, and removable appliances, some articles studied multiple types of appliances. In vitro studies consistently demonstrated significant reductions in bacterial adhesion and biofilm formation across all appliance types, with silver-based coatings and nitrogen-doped TiO_2_ showing the broadest evidence base; combination systems (Ag/ZnO, CuO-ZnO) outperformed individual agents. The 13 in vivo and clinical studies provided limited but directionally supportive evidence: silver nanoparticle-incorporated acrylic retainers reduced *S. mutans* counts in a double-blind RCT, and silver-infiltrated tungsten material-maintained biofilm reduction after simulated two-year abrasion. Coating durability emerged as an important determinant of potential clinical translation. Surface-deposited thin-film coatings degraded substantially within one month of intraoral use, whereas substrate-integrated approaches showed greater longevity. This scoping review maps the current evidence landscape, identifies coating durability and clinical endpoint validation as critical gaps, and prioritizes silver-based and nitrogen-doped TiO_2_ coatings for future randomized clinical trials.

## 1. Introduction

Orthodontic treatment introduces a well-recognized clinical challenge: fixed and removable appliances impair oral hygiene by acting as retention sites for dental plaque, promoting biofilm accumulation that can cause lasting damage to dental and periodontal tissues [1,2]. The most clinically relevant complication is the development of white spot lesions (WSLs), early signs of enamel demineralization affecting between 30% and 90% of orthodontic patients [3,4,5,6,7]. These lesions result from acid-producing bacteria, principally *Streptococcus mutans* and *Lactobacillus* species, colonizing bracket margins and lowering local pH below the critical threshold for enamel dissolution. Beyond cariogenic damage, orthodontic appliances also harbor periodontal pathogens such as *Porphyromonas gingivalis* and *Aggregatibacter actinomycetemcomitans*, contributing to gingival inflammation and potential long-term periodontal complications [3,4,5,6,7]. Current prevention relies on patient-dependent strategies, such as brushing, flossing, fluoride application, and mouth rinses, that are limited by variable compliance and provide only transient protection, as bacterial recolonization of appliance surfaces resumes rapidly [7,8]. This limitation has driven growing interest in material-based, passive preventive strategies that function independently of patient behavior.

Antibacterial surface modifications and coating strategies modify or structurally alter the appliance surface to resist bacterial colonization at the critical adhesion interface. Strategies investigated to date span four broad categories: surface-deposited thin-film coatings, metal-based systems (silver nanoparticles, zinc oxide, titanium dioxide, copper oxide) acting through ion release or light-activated mechanisms, and polymer-based systems (polyethylene glycol, chitosan) creating low-adhesion surfaces; plasma-based and ion-implantation surface treatments (plasma polymerization, plasma immersion ion implantation and deposition, PIIID), which modify surface chemistry at the nanoscale without a discrete deposited layer; bulk-incorporated antibacterial agents distributed throughout the appliance matrix material (e.g., AgNPs in PMMA acrylic resin, silver-infiltrated tungsten alloy); and non-release surface modifications that alter surface chemistry without introducing exogenous agents [9]. Combination coatings (Ag/ZnO, CuO-ZnO, N-doped TiO_2_, TiO_2_:Ag) have consistently outperformed single-agent counterparts in vitro [10,11,12,13,14,15,16,17]. Several narrative reviews have summarized in vitro evidence for specific coating classes, including silver nanoparticles, zinc oxide, and functional archwire coatings [10,11,12,13,14,15,16,17]. However, these reviews are restricted to single material categories, do not distinguish experimental, preclinical, and clinical evidence levels, and do not systematically map the translational gap between laboratory efficacy and intraoral performance across the full range of orthodontic appliance types.

The central unresolved question is whether in vitro antibacterial efficacy translates to clinically meaningful protection under real intraoral conditions. In the oral environment, photocatalytic light dependence, salivary protein fouling, mechanical loading from archwire sliding and chewing forces, and polymicrobial biofilm complexity substantially attenuate coating performance in ways that simplified laboratory models do not capture. Additional unaddressed challenges include the limited evaluation of sterilization effects on coating integrity, the predominance of single-species and short-duration models, inconsistent outcome reporting across studies, and the near-total absence of clinical endpoints such as WSL incidence or periodontal health measured over full treatment duration. There is a need to characterize this translational gap across all appliance types and identifying which coating categories progressed beyond in vitro proof-of-concept.

The objective of this scoping review is to comprehensively map the existing evidence on antibacterial surface modifications and coating strategies for orthodontic appliances across all device types, characterize the range of materials, mechanisms, and testing methodologies employed, and assess the current state of clinical translation. Given the heterogeneity of coating technologies, orthodontic substrates, bacterial models, and outcome measures, a scoping review methodology is appropriate to systematically chart this emerging field and identify the gaps that must be addressed before definitive efficacy meta-analyses can be conducted.

## 2. Materials and Methods

### 2.1. Protocol and Registration

This review was conducted in accordance with the PRISMA Extension for Scoping Reviews (PRISMA-ScR) guidelines [18] and the methodological framework proposed by Arksey and O’Malley [19] and refined by Levac et al. [20]. PROSPERO (Prospective Register of Systematic Reviews) does not accept scoping review registrations; its eligibility criteria explicitly restrict registration to systematic reviews of interventions with direct health-related outcomes in humans or animals [21] (Centre for Reviews and Dissemination, University of York. PROSPERO eligibility criteria [Internet; accessed 2023]. Available at: www.crd.york.ac.uk/prospero (accessed on 20 August 2026)). Accordingly, this scoping review was not registered in PROSPERO. The search strategy, eligibility criteria, data extraction framework, and synthesis approach were defined a priori and documented before screening commenced. The a priori protocol is available from the corresponding author upon reasonable request. Future scoping reviews in this field may consider registration in the Open Science Framework, a free, open-source platform managed by the Center for Open Science that supports pre-registration of diverse study types, including scoping review protocols, and provides a time-stamped, publicly accessible record of pre-specified methods (Center for Open Science, 2011; available at: osf.io). However, the search strategy, inclusion criteria, and data extraction framework were defined and documented before screening commenced to ensure methodological rigor and reproducibility.

The scoping review methodology was selected over a traditional systematic review with meta-analysis for several critical reasons. The field encompasses highly heterogeneous study designs (in vitro materials science experiments, animal models, clinical trials), coating technologies (metallic nanoparticles, photocatalytic oxides, polymeric systems, composite materials), orthodontic substrates (brackets, archwires, aligners, bands, miniscrews, elastomeric ligatures, removable appliances), bacterial models (single-species reference strains, multi-species biofilms, clinical isolates, in vivo oral microbiome), and outcome measures (colony-forming unit enumeration, zone of inhibition, biofilm biomass quantification, viability staining, microscopy, molecular assays). This diversity precludes meaningful meta-analysis and pooled effect-size estimation.

### 2.2. Eligibility Criteria

The eligibility criteria were defined according to the PICO framework to ensure a structured and transparent study selection process. The research question was formulated as follows: Population (P): orthodontic appliances intended for intraoral use; Intervention (I): antibacterial surface coatings, surface modifications, or bulk incorporation of antibacterial agents; Comparison (C): uncoated or conventionally treated orthodontic materials; and Outcomes (O): antibacterial performance, assessed through microbiological, imaging, or clinical outcome measures.

Original in vitro, in vivo, and clinical studies published in English were eligible if they evaluated the antibacterial efficacy of coated or surface-modified orthodontic appliances compared with an appropriate control. Studies focusing exclusively on orthodontic adhesives, cements, or non-orthodontic dental materials, as well as reviews, editorials, case reports, conference abstracts, and studies lacking antibacterial outcomes or an appropriate control group, were excluded.

A detailed description of the inclusion and exclusion criteria, including the full PICO framework and eligibility definitions, is provided in the Supplementary Materials.

### 2.3. Information Sources and Search Strategy

#### 2.3.1. Electronic Databases

A comprehensive literature search was conducted across five electronic databases:

- PubMed/MEDLINE (National Library of Medicine)
- Scopus (Elsevier)
- Web of Science Core Collection (Clarivate Analytics)
- Cochrane Central Register of Controlled Trials (CENTRAL) (Cochrane Library)
- Google Scholar (first 200 results ranked by relevance)

The initial search was performed in December 2025, with a final update conducted in May 2026 to capture recently published studies.

#### 2.3.2. Search Strategy

The search strategy combined three concept blocks using Boolean operators: (1) orthodontic appliances, (2) antibacterial agents and coatings, and (3) antibacterial outcomes. Both Medical Subject Headings (MeSH) terms and free-text keywords were used to maximize sensitivity. The full search strategy for PubMed is presented in the Supplementary Materials.

### 2.4. Study Selection Process

#### Screening Phases

Study selection was conducted in three sequential phases: (1) removal of duplicate records using Covidence with additional manual verification; (2) independent title and abstract screening by two reviewers (B.F.-A. and D.N.J.); and (3) independent full-text assessment of potentially eligible studies against the predefined eligibility criteria. Disagreements at any stage were resolved through discussion, with a third reviewer (A.P.-P.) consulted when consensus could not be reached.

Inter-rater agreement between reviewers was assessed using Cohen’s kappa (κ) statistic, with agreement interpreted according to the Landis and Koch classification. Further details regarding the screening process and reviewer agreement are provided in the Supplementary Materials.

### 2.5. Data Collection Process

#### 2.5.1. Data Extraction

Data extraction was performed independently by two reviewers (B.F.-A. and D.N.J.) using a standardized, pilot-tested data extraction form created in Microsoft Excel (Microsoft Corporation, Redmond, WA, USA). The extraction form was pilot-tested on five randomly selected included studies and refined to ensure clarity and completeness. Discrepancies in extracted data were resolved through discussion and re-examination of the source article, with arbitration by the third reviewer (A.P.-P.) when necessary.

#### 2.5.2. Data Items

Data were systematically extracted using a standardized form that included study characteristics, orthodontic appliance and material type, coating/intervention characteristics, antibacterial testing conditions, and reported outcomes. Extracted outcomes comprised antibacterial efficacy, biocompatibility, durability, and other relevant quantitative and qualitative findings. When necessary, additional data were obtained from graphical representations or by contacting the corresponding authors. A detailed description of all extracted variables is provided in the Supplementary Materials.

### 2.6. Methodological Characterization and Evidence Quality Appraisal

Consistent with established scoping review methodology [22], no formal risk-of-bias assessment was applied to included studies. Formal bias assessment instruments validated for clinical trials (Cochrane Risk of Bias 2.0, ROBINS-I) and animal studies (SYRCLE’s RoB tool) evaluate constructs such as randomization, allocation concealment, blinding, and selective outcome reporting, domains that are structurally inapplicable to in vitro materials characterization experiments, which constitute 88% of the evidence base in this review [19]. No consensus framework for formal bias scoring in laboratory-based antibacterial coating research has been established or validated, and applying clinical trial tools to bench science would yield uninterpretable scores that misrepresent the nature of the evidence.

However, to enable readers to evaluate the methodological robustness and translational relevance of included studies, a structured methodological characterization was applied to all 109 studies using a pre-defined extraction framework. This approach, recommended by Peters et al. [22] for scoping reviews in health-related fields, provides transparent reporting of the factors most likely to influence the reliability and generalizability of findings without imposing inappropriate quality hierarchies on a heterogeneous evidence base. Methodological characteristics extracted are reported in the Supplementary Materials.

### 2.7. Biological Relevance Hierarchy and Evidence Classification Framework

To enable systematic comparison of findings across studies with markedly different experimental designs, a four-tier biological relevance hierarchy was applied to classify the translational strength of each included study. This framework, adapted from frameworks proposed for preclinical antimicrobial research [23], classifies studies based on four domains that collectively determine how closely the experimental conditions approximate the clinical orthodontic environment. The biological relevance hierarchy and evidence classification framework are reported in the Supplementary Materials.

### 2.8. Evidence Synthesis

As a scoping review, this study does not apply formal certainty-of-evidence frameworks such as GRADE (Grading of Recommendations Assessment, Development and Evaluation), which are designed for systematic reviews evaluating intervention efficacy and require homogeneous outcome measures amenable to meta-analysis [24]. Evidence was synthesized narratively and organized according to orthodontic appliance type, coating material, and study design (in vitro, in vivo, and clinical studies).

The synthesis summarizes the reported antibacterial effects, methodological characteristics, durability, and biocompatibility of the evaluated coatings, while highlighting similarities, inconsistencies, and knowledge gaps across the literature. Given the exploratory nature of scoping reviews, no weighting of studies based on methodological quality or calculation of pooled effect estimates was undertaken (described in the Supplementary Materials).

### 2.9. Data Synthesis and Analysis

Due to substantial heterogeneity in study designs, orthodontic appliances, coating types, bacterial species, testing methods, and outcome measures, a narrative synthesis was deemed most appropriate. Meta-analysis was not feasible due to the diversity of interventions and outcomes, inconsistent reporting of quantitative data, and the predominance of in vitro studies with non-standardized protocols. The narrative synthesis was conducted according to the Synthesis Without Meta-analysis (SWiM) reporting guideline [25]. Synthesis Structure is reported in the Supplementary Materials.

### 2.10. Reporting and Transparency

**Figure 1:**
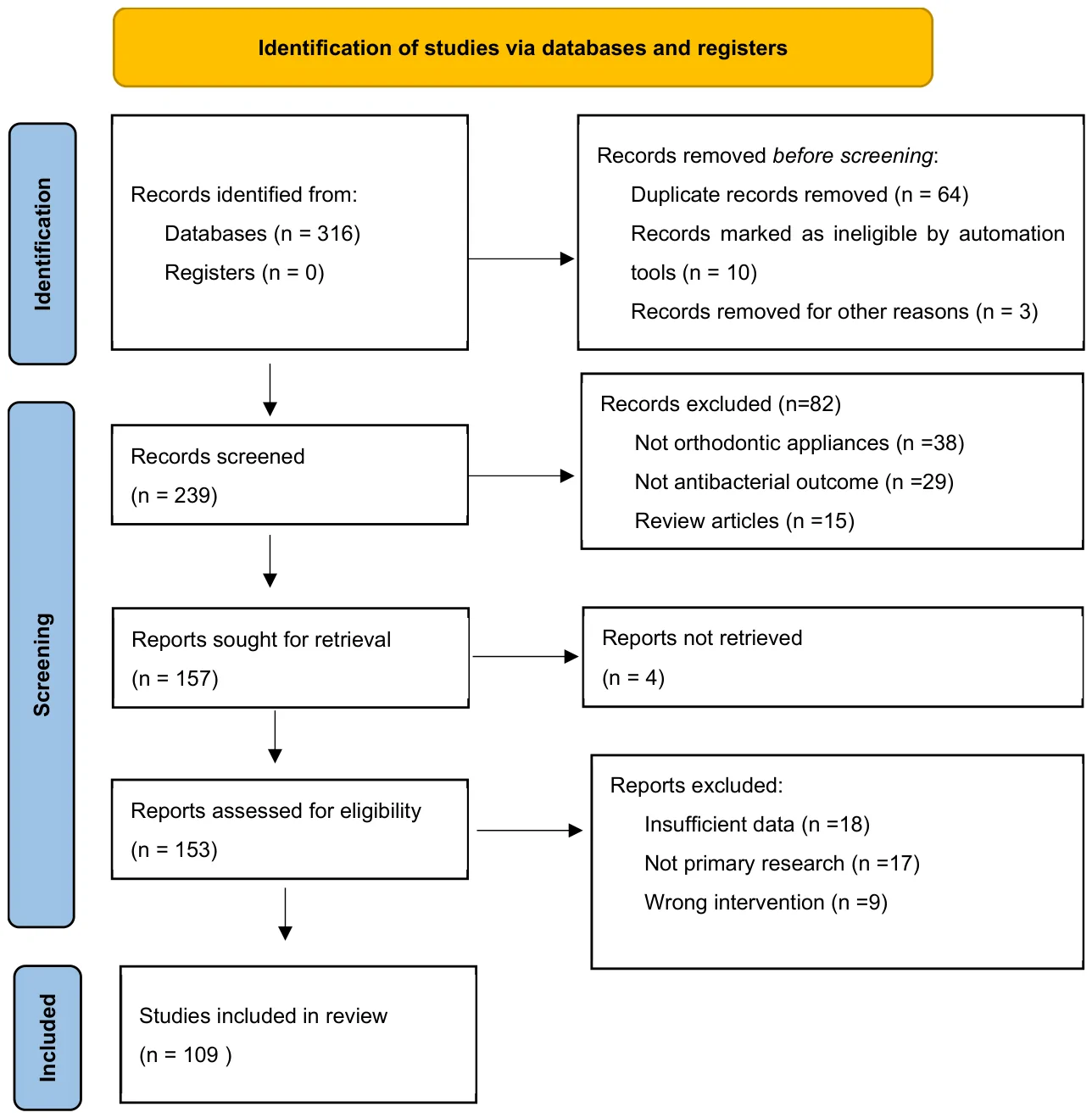
Study selection process, PRISMA-ScR flowchart.

This scoping review is reported in accordance with the Preferred Reporting Items for Systematic Reviews and Meta-Analyses extension for Scoping Reviews (PRISMA-ScR) checklist. A completed PRISMA-ScR checklist is provided in the Supplementary Materials. The study selection process is documented in a PRISMA-ScR flow diagram presented in the Section 3 (Figure 1). Any deviations from the registered protocol were documented and justified in the Results or Discussion sections.

Generative artificial intelligence tools Research Rabbit (web-based AI tool, 2024 version, ResearchRabbit Inc., Seattle, WA, USA. Available at: www.researchrabbit.ai) was used for literature discovery, Elicit (AI research assistant, 2024 version, developed by Ought Inc. (San Francisco Bay Area, CA, USA)) for rapid literature screening, and SciSpace Research Agent (AI-powered research assistant, version 2025, developed by Typeset.io) for manuscript preparation. No AI tools were used for study selection, data extraction, risk of bias assessment, or interpretation of results. All AI-generated text was reviewed, edited, and verified by the authors for accuracy and appropriateness. The authors take full responsibility for the content of this manuscript.

## 3. Results

### 3.1. Selection of Sources of Evidence

The literature search identified 316 records, of which 239 remained after duplicate removal and initial screening in Covidence. Following title/abstract screening and full-text assessment, 109 studies met the eligibility criteria and were included in the qualitative synthesis, comprising 96 in vitro studies, 8 in vivo animal studies, and 5 clinical studies (Figure 1). Reasons for full-text exclusion are summarized in the PRISMA-ScR flow diagram.

Study selection was performed independently by two reviewers, with disagreements resolved by discussion or consultation with a third reviewer when necessary. Inter-rater agreement was initially assessed using Cohen’s kappa; however, final κ values could not be calculated retrospectively because Covidence does not retain individual reviewer decisions after conflict resolution.

### 3.2. Characteristics of Sources of Evidence

The 109 included studies were published between 2007 and 2026, with 74 (67.9%) published during the last five years (2020–2026), reflecting the growing interest in antibacterial surface coatings for orthodontic applications. The studies originated from 28 countries, with the highest research output from China (n = 24), followed by India (n = 18), Iran (n = 14), and the United States (n = 11). Regarding study design, 96 studies (88.1%) were conducted in vitro, 8 (7.3%) were in vivo animal studies, and only 5 (4.6%) were clinical investigations, comprising three randomized controlled trials and two split-mouth controlled trials. The distribution of studies according to orthodontic appliance type is presented in Table 1. Brackets were the most frequently investigated appliance (n = 37), followed by archwires (n = 34), miniscrews (n = 14), aligners and acrylic removable appliances (n = 10), elastomeric ligatures (n = 5), and orthodontic bands (n = 2). Seven studies evaluated more than one appliance type.

Silver-based coatings were the most extensively investigated antibacterial strategy (n = 45), followed by titanium dioxide (TiO_2_)-based coatings (n = 26), zinc oxide (ZnO)-based coatings (n = 17), hybrid or composite coatings (n = 15), and polymer-based coatings, including chitosan and quaternary ammonium compounds (n = 12).

*Streptococcus mutans* was the most frequently evaluated microorganism (n = 89), followed by *Staphylococcus aureus* (n = 24), *Lactobacillus acidophilus* (n = 18), *Escherichia coli* (n = 16), *Porphyromonas gingivalis* (n = 12), and *Candida albicans* (n = 10). Multi-species biofilm models were used in 14 studies, although single-species models predominated.

Colony-forming unit (CFU) enumeration was the most reported antibacterial outcome (n = 76), followed by scanning electron microscopy (SEM) evaluation of bacterial adhesion (n = 64), biofilm biomass quantification (n = 42), zone of inhibition (ZOI) assays (n = 38), and live/dead viability staining (n = 28). Most studies (n = 71) employed multiple complementary outcome measures.

### 3.3. Methodological Characteristics of Included Studies

Consistent with scoping review methodology, no formal risk-of-bias assessment was conducted. Instead, a structured methodological characterization framework (described in Section 2.6) was applied to all 109 studies to enable transparent evaluation of translational relevance and methodological robustness.

#### 3.3.1. Methodological Characteristics of In Vitro Studies (n = 96)

Bacterial model complexity: Most in vitro studies (n = 78) employed single-species reference strains (*S. mutans* ATCC 25175 or *S. aureus* ATCC 6538), which provide proof-of-concept antibacterial activity but do not replicate the polymicrobial ecology of the orthodontic oral environment. Fourteen studies used multi-species biofilm models, and only 4 studies employed salivary inoculum, the most clinically relevant bacterial challenge model.

Biofilm maturation stage: Forty-two studies tested coatings against planktonic bacterial suspensions (≤24 h), 37 studies used early biofilms (24–48 h), 15 studies tested mature biofilms (48–72 h), and only 2 studies evaluated extended biofilms (>72 h). Planktonic assays may overestimate antibacterial performance compared with mature biofilm models because they do not reproduce extracellular matrix protection and ecological complexity.

Coating characterization completeness: Sixty-four studies reported surface morphology characterization (SEM/AFM), 58 studies reported elemental composition (EDS/XPS), 42 studies reported coating thickness measurements, 38 studies reported crystalline structure (XRD), 24 studies reported adhesion strength testing, and 31 studies reported ion release kinetics. Only 28 studies reported ≥4 of these parameters, indicating adequate physicochemical characterization.

Durability assessment: Most in vitro studies (n = 64) tested coatings only in the as-prepared state without any durability challenge. Twenty-two studies included post-aging simulation (thermocycling, pH cycling, artificial saliva immersion), 8 studies performed mechanical abrasion testing, and only 2 studies included simulated long-term intraoral exposure (>6 months equivalent).

Statistical robustness: Seventy-eight studies reported ≥3 independent replicates with inferential statistics (*t*-tests, ANOVA, or non-parametric equivalents). Eighteen studies reported inadequate replication (n < 3) or lacked inferential statistical analysis.

#### 3.3.2. Methodological Characteristics of In Vivo and Clinical Studies (13)

Among the clinical evidence, three studies were randomized controlled trials and two employed a split-mouth controlled design, while eight studies were conducted in animal models. Only two of the in vivo and clinical studies reported an a priori sample size calculation. The median sample size in the clinical studies was 24 participants (range: 12–40). Follow-up periods were generally short, with six studies evaluating outcomes for up to four weeks, six reporting follow-up between one and three months, and only one extending beyond three months (75 days in an animal model). None of the included clinical studies exceeded 12 weeks of follow-up, and no study assessed the long-term performance of antibacterial coatings throughout a complete orthodontic treatment.

The clinical studies primarily evaluated surrogate microbiological outcomes, including *Streptococcus mutans* colony-forming unit counts, real-time PCR cycle threshold (Ct) values, and total biofilm volume. None used the incidence of white spot lesions or caries progression as the primary endpoint. Only one study additionally assessed enamel calcium and phosphorus content as a secondary outcome, providing indirect evidence of a potential caries-preventive effect. Methodological safeguards were inconsistently reported. Participant blinding was described in two studies, operator blinding in one, outcome assessor blinding in three, allocation concealment in two, and intention-to-treat analysis in one. Most studies reported a loss to follow-up below 20%.

#### 3.3.3. Biological Relevance Tier Distribution

This distribution indicates that 73% of the evidence base (Tiers 1–2) drives from preclinical models with limited translational relevance. Tier 4 evidence is available only for silver-based formulations on N-doped TiO_2_ brackets [12], nanosilver-coated stainless steel brackets evaluated for the prevention of enamel demineralization in orthodontic patients [26], PMMA appliances [27,28], TiO_2_-coated stainless steel wires [29], and nanosilver-coated brackets assessed for in vivo antibacterial activity and silver ion release [30]. All other coating categories (ZnO, CuO, chitosan, graphene oxide, quaternary ammonium compounds, hybrid systems) lack clinical or in vivo validation. A detailed description of the tier distribution is provided in the Supplementary Materials.

#### 3.3.4. Summary of Methodological Quality Signals

The structured characterization revealed several patterns that inform interpretation of reported efficacy values:

- Studies using single-species planktonic assays reported systematically higher antibacterial efficacy (reported CFU reduction range across studies: 85–99%) than studies using mature multi-species biofilms or salivary inoculum models (reported CFU reduction range across studies: 40–70%), suggesting that published efficacy ranges overestimate real-world performance.
- Only 33% of in vitro studies (n = 32/96) included any form of durability assessment beyond the as-prepared state, limiting conclusions about long-term clinical performance.
- No included clinical study evaluated white spot lesion incidence or caries increment as a primary outcome, precluding direct assessment of clinical effectiveness for caries prevention.
- The median follow-up duration for clinical studies was 6 weeks (range: 2–12 weeks), far shorter than typical orthodontic treatment duration (18–24 months).

### 3.4. In Vitro Studies

#### 3.4.1. Orthodontic Archwires

Thirty-four in vitro studies evaluated antibacterial coatings on orthodontic archwires, predominantly testing silver-based agents, TiO_2_-based coatings, and ZnO nanoparticles on NiTi and stainless-steel substrates against *Streptococcus mutans*. The highest efficacies were observed for ZnO nanoparticles via CVD [27], electrodeposited AgNPs on NiTi [28], N-doped TiO_2_ under visible light [29], and TiO_2_:Ag coatings [30]. Dose-response relationships were consistently demonstrated across coating types [31,32]. Nanoparticle size and morphology significantly influenced performance, with smaller AgNPs outperforming larger particles [33] and CVD-deposited ZnO outperforming electrospun fibers [27].

Durability data were sparse and limited to short time points. The longest documented effectiveness was 42 days for polydopamine/PEDOT/AgNPs systems [31], followed by 28 days for TiN-Cu [32] and CHX-HMP nanoparticle coatings [33]. TiO_2_:Ag coatings showed declining efficacy over time, consistent with depletion of releasable silver [29]. Only two studies tested coatings under simulated mechanical stress [34,35].

Biocompatibility was acceptable for most coatings tested. TiN-Cu coatings showed the highest relative cell viability in 28-day eluate testing [32], N-doped TiO_2_ exhibited zero cytotoxicity through 120 h [36], and silver-containing coatings released Ag^+^ ions below harmful thresholds [31,37]. Dose-dependent toxicity was identified for ZnO nanoparticles at higher concentrations [35]. Sixteen of 34 studies did not include any cytotoxicity assessment.

Silver-based coatings achieved robust bactericidal effects through Ag^+^ ion release but showed self-limiting efficacy as silver depleted [29,38]. TiO_2_-based photocatalytic coatings required UV illumination, limiting intraoral applicability [36]. ZnO coatings combined contact killing with ROS generation, with efficacy varying by deposition method [39]. Antifouling coatings such as PEG produced modest reductions through steric repulsion rather than bactericidal mechanisms [40]. Balancing efficacy, duration, and safety, the polydopamine/PEDOT/AgNPs system maintained effectiveness for 42 days with acceptable biocompatibility [31], and TiN-Cu coatings offered sustained efficacy with the highest cell viability and reduced Ni ion release [32]. All evidence remains preclinical, with simplified monoculture models, small sample sizes, absence of blinding, and heterogeneous bacterial quantification methods limiting clinical translation.

#### 3.4.2. Orthodontic Brackets

Thirty-seven in vitro studies evaluated antibacterial coatings on orthodontic brackets, with 34 of 37 reporting statistically significant reductions in bacterial adhesion and biofilm formation. The highest efficacies were observed for nitrogen-doped TiO_2_ coatings under visible light [12], TiO_2_-coated ceramic brackets under UV-A [41], ZnO/carbon quantum dot composites under natural light [14], and CuO-based coatings [11]. Silver-based coatings showed variable efficacy but functioned independently of light activation [42]. Combination coatings consistently outperformed single-agent systems [10].

The longest sustained antibacterial effects were reported for N-doped TiO_2_ coatings [12], nanoparticle combinations of Ag, ZnO, and Ag/ZnO [10], and CuO, TiO_2_, and HA-Ag coatings [43]. Silver-infiltrated tungsten material uniquely demonstrated retention of antibacterial properties after simulated 2-year toothbrush abrasion [44]. Many studies were limited to 24–48 h evaluations; only seven evaluated beyond 30 days, a notable limitation given typical orthodontic treatment duration.

Biocompatibility testing was conducted in only a minority of studies. N-doped TiO_2_ thin films showed the most comprehensive safety profile, including grade 0 cytotoxicity and absence of mucosal irritation, systemic toxicity, hemolysis, and genotoxicity [45]. One in vitro study reported TiO_2_ crystalline phase: rutile phase showed severe cytotoxicity, whereas anatase phase maintained acceptable viability [46]; however, this finding cannot be generalized, as TiO_2_ cytotoxicity is highly dependent on particle size, surface chemistry, dose, agglomeration state, illumination, and cell type. Silver-based coatings were generally biocompatible at tested concentrations [47]. Ion release from ZnO and CuO coatings remained below toxic thresholds throughout 28-day evaluation [48]. Approximately 22 of 37 studies did not conduct formal cytotoxicity testing.

Light-dependent versus light-independent coating mechanisms explained much of the apparent heterogeneity. Standard TiO_2_ required UV-A illumination, offering uncertain clinical utility given limited intraoral UV exposure [41]. Nitrogen-doped TiO_2_ shifted photocatalytic activation into the visible light spectrum with sustained activity over 90 days [12]. Silver-based coatings functioned through ion release independent of light, making antibacterial activity more predictable in the oral cavity [42].

The single negative result, titanium nitride and TN + calcium phosphate coatings showing no significant reduction in *S. mutans* biofilm [49], appears attributable to the absence of photocatalytic ROS-generating properties. Among the investigated materials, N-doped TiO_2_ [12,45] demonstrated a favorable balance of antibacterial activity and reported biocompatibility under laboratory conditions [46]; however, clinical validation remains lacking. Silver-based combination coatings provided strong efficacy with sustained activity and acceptable safety profiles [10,48]. All evidence derives from in vitro monospecies models with predominantly short evaluation periods, and no study evaluated these coatings in a clinical setting.

#### 3.4.3. Orthodontic Bands

Two in vitro studies investigated silver and zinc oxide nanoparticle coatings on stainless steel orthodontic bands. Silver nanoparticle coatings demonstrated the strongest antimicrobial activity, achieving ≥2-log_10_ reduction against *S. mutans*, *L. acidophilus*, and *C. albicans*, while zinc oxide coatings reached this threshold only for *S. mutans* [50]. Both coating types maintained cell viability above 80%, with nano-ZnO showing slightly higher viability (91.8%) compared to nano-Ag (85.7%). Nano-ZnO was considered more favorable when balancing efficacy and safety due to lower toxicity and adequate activity against the primary cariogenic pathogen, whereas nano-Ag offered broader-spectrum antimicrobial coverage at a modest biocompatibility cost [50].

Durability data were limited: physical coating retention was confirmed after 30 days of simulated brushing in one study, but antimicrobial activity was not re-quantified over time in either study [50,51]. The two studies used different deposition techniques (electrostatic spray-assisted vapor deposition versus thermal evaporation) and different nanoparticle size ranges (average 20 nm versus 45–60 nm), limiting direct cross-study comparison [50,51]. Neither study reported randomization or blinding procedures. The question of long-term antimicrobial durability remains unanswered, and no in vivo validation has been conducted.

#### 3.4.4. Orthodontic Ligatures

Five in vitro studies evaluated antibacterial coatings on elastomeric ligatures and modules, testing silver-based agents, chlorhexidine-releasing systems, and metal oxide nanoparticles. Quantitative bacterial reductions ranged from 57% to 96%, with the highest efficacies observed for nano silver fluoride elastomeric modules (NSF-EP2: 57% CFU reduction, 86% biofilm thickness reduction, 96% live/dead cell ratio reduction over 7 days) [52] and chlorhexidine-releasing elastomeric ligatures (sustained CHX release at 1 µg/mL for 48 h) [53]. Silver nanofilm coatings on elastomeric ligatures were effective, while bismuth nanofilm coatings showed no significant *S. mutans* reduction [54], representing one of the few negative results in the ligature literature.

Sustained-release coatings showed mechanistically distinct behavior. CHX-releasing elastomeric ligatures demonstrated sustained CHX release for 48 h [55], while NSF-EP2 modules maintained antibacterial activity for 7 days [52]. However, the fundamental limitation of sustained-release approaches is progressive depletion of the active agent, as demonstrated in other device categories [56]. Biocompatibility data were limited, with only one study reporting formal cytotoxicity assessment [52].

The evidence base for ligature coatings is substantially smaller than for brackets or archwires, with heterogeneous coating types, short evaluation periods (predominantly 24–48 h), and no clinical or in vivo validation. The clinical relevance is further limited by the fact that elastomeric ligatures are typically replaced at monthly adjustment appointments, requiring only short-term antibacterial activity.

#### 3.4.5. Orthodontic Miniscrews

Fifteen in vitro studies evaluated antibacterial coatings on titanium or titanium alloy orthodontic miniscrews, encompassing silver-based nanocomposites, zinc oxide nanoparticles, chitosan-antibiotic combinations, hydroxyapatite composites, TiO_2_ photocatalytic coatings, PEG, and selenium nanoparticles. The most frequently tested organisms were *S. mutans* and *S. aureus*. No clinical or animal model validation of antibacterial outcomes was identified in this subsection.

All studies reported superior antibacterial performance for coated substrates compared to uncoated controls, though the magnitude of effect varied by coating type, delivery system, and target organism. The largest reductions were achieved by Ag/a-C:H nanocomposites, with a clear dose-response relationship [53]. In head-to-head comparisons, ZnO nanoparticles via electrochemical deposition showed the highest antimicrobial activity against *S. aureus* and superior cytocompatibility relative to Ag/HA nanoparticles [57]. ZnO/doxycycline-loaded TiO_2_ nanotubes achieved the largest inhibition zone against *P. gingivalis* [58], while chitosan-AgNP [59] and HAP/chitosan coatings [60] showed concentration-dependent increases in zone of inhibition. PEG plasma coatings operated through a distinct antifouling mechanism involving steric repulsion rather than bactericidal activity [61].

A critical determinant of efficacy was the coating delivery system rather than the antibacterial agent alone. Direct AgNP deposition produced no antibacterial effect, whereas the same agent delivered via biopolymer carrier generated clear inhibition zones against all tested species [62]. Similarly, unloaded TiO_2_ nanotubes showed no antimicrobial activity, whereas nanotubes loaded with ZnO and doxycycline maintained activity for 30 days [58]. Bacterial strain specificity was also evident: ZnO-based coatings showed strong activity against Gram-positive organisms but limited efficacy against *E. coli* [63], whereas Ag/a-C:H nanocomposites were more effective against *E. coli* than *S. aureus* [64]. Surface roughness emerged as a confounding variable: integration of CaP into AgNP coatings paradoxically promoted microbial adhesion despite the presence of antibacterial agents [65].

Most studies measured outcomes at 24–48 h only. Among studies assessing longer time points, ZnO/doxycycline-loaded nanotubes maintained activity for 30 days with progressive decline [58], and chitosan-azithromycin coatings delivered antibiotic for approximately 7 days with continued but declining release until week 4 [55].

Only 6 of 14 studies included biocompatibility evaluation. Dose-dependent cytotoxicity was consistently observed for silver-based coatings, with improved cell proliferation at lower concentrations but potential cytotoxicity at higher loadings [59,64]. Coating retention during miniscrew insertion was assessed by only two studies: one reported partial detachment during simulated implantation [66], and another demonstrated that autoclave sterilization caused nanoparticle agglomeration and complete loss of antibacterial activity [63]. The TiO_2_ photocatalytic approach achieved near-zero CFU rapidly but requires continuous UV irradiation, limiting intraoral applicability [54]. Selenium nanoparticles showed comparable antibacterial activity to silver with lower cytotoxicity [67], but evidence is limited to a single study. Collectively, in vitro antibacterial efficacy has not been validated under conditions representative of clinical use.

#### 3.4.6. Orthodontic Acrylic and Clear Appliances

Ten in vitro studies evaluated antibacterial coatings on acrylic resin removable appliances and clear aligners, testing gold nanoparticle-based coatings, TiO_2_ under UVA irradiation, ZnO and MgO combinations, curcumin-based approaches, silver-loaded PMMA, and polysaccharide-based coatings. The highest efficacies were observed for QA-GNCs on clear aligners [68] and TiO_2_ under UVA irradiation on acrylic resin [68]. ZnO + MgO combination coatings outperformed individual ZnO or MgO coatings [28]. Curcumin-based approaches demonstrated clear dose-response relationships [69].

The longest reported duration was 3 months for QA-GNC-coated aligners [70]. TiO_2_ coating demonstrated mechanical durability for approximately 2 years, though its antibacterial mechanism requires continuous UVA irradiation [68]. Silver-loaded PMMA showed dramatic durability failure, with biofilm coverage effectively returning to control levels after a 6-month washout [56]. MSN-based drug delivery systems offered intermediate solutions with sustained antifungal effects up to 28 days [71].

Biocompatibility was comprehensively assessed in only two studies involving gold-based nanoparticle coatings, which demonstrated excellent profiles with no hemolysis, mucosal irritation, or cytotoxicity [70]. A consistent trade-off between antimicrobial loading and mechanical integrity emerged for curcumin-based studies: concentrations providing meaningful antibacterial activity maintained flexural strength above the ISO-required minimum, but higher loadings resulted in mechanical failure [69,72].

The divergence in duration of antibacterial effectiveness is largely explained by coating application method and mechanism. Surface coatings applied by electrostatic self-assembly demonstrated strong, contact-dependent bactericidal activity with minimal agent release, preserving coating longevity [70]. By contrast, systems relying on agent release inherently lose effectiveness as the active agent depletes [56,69,71]. Substrate type also influences applicability: clear aligners are typically replaced every 1–2 weeks, whereas acrylic retainers are worn for months or years, making long-term durability essential [73].

### 3.5. Clinical and In Vivo Evidence

Thirteen studies evaluated antibacterial coatings on orthodontic materials in clinical, in vivo, or in situ settings, testing silver-based modifications (nanosilver, silver ion implantation, silver infiltration, silver nanoparticles in acrylic), titanium-based coatings (TiO_2_, N-doped TiO_2_, TiN), polyethylene glycol plasma coatings, and gold nanoparticle coatings. Results are shown in Table 2. Study designs ranged from randomized clinical trials and split-mouth designs in human patients to in situ splint models and animal studies, with follow-up durations varying from 48 h to 75 days. Sample sizes in human clinical studies ranged from 12 to 68 patients, with most employing split-mouth or crossover designs to reduce inter-patient variability.

All studies testing purpose-designed antibacterial coatings reported beneficial effects in reducing bacterial adhesion or biofilm formation relative to uncoated controls, though the magnitude and duration varied substantially. The strongest clinical evidence comes from the Farhadian et al. randomized controlled trial (n = 61), which demonstrated a mean difference of 40.31 CFU (95% CI: 24.83–55.79, *p* < 0.001) favoring silver nanoparticle-containing retainers at 7 weeks [27]. Hashem et al. confirmed enamel protection over 2 months in a parallel-group RCT of 32 patients, with stable mineral content in the coated group and significant decreases in the uncoated group [26], providing the only direct clinical evidence of downstream caries-preventive effects. The two in situ splint studies (Meyer-Kobbe et al., Denis et al.) demonstrated 60–78% biofilm volume reduction with silver-infiltrated tungsten material even after simulated 2-year toothbrushing abrasion [44,74], with PIIID silver ion implantation uniquely achieving a significant increase in the proportion of dead bacteria [74].

A critical practical consideration is whether coatings maintain efficacy over clinically relevant timeframes. The evidence reveals a consistent pattern of diminishing effectiveness over time for surface-deposited coatings. N-doped TiO_2_ coating on brackets showed better efficacy at 30 days than at 60 days [75] and TiO_2_ coating on NiTi archwires lost 60% of its thickness after one month due to delamination and deterioration [80]. By contrast, coatings that penetrate or are structurally integrated into the substrate demonstrated greater durability: silver-infiltrated tungsten material maintained antibacterial properties after abrasion simulating two years of toothbrushing [44], PIIID silver ion implantation was specifically highlighted for improved abrasion resistance [74], and silver nanoparticles incorporated into acrylic retainer material showed sustained antimicrobial effects over 7 weeks [27].

No study reported clinically significant adverse effects attributable to antibacterial coatings. Metin-Gürsoy et al. found similar inflammatory responses between nanosilver-coated and standard brackets when implanted subcutaneously in rats, though brown-black granules of uncertain significance were observed in the nanosilver group [79]. Salivary and serum silver concentrations were elevated on Day 7 in the nanosilver-coated bracket group but remained far below thresholds associated with argyria [30]. Multiple authors noted that comprehensive long-term toxicity data are still lacking for clinical translation.

The most important source of heterogeneity is the distinction between surface-deposited coatings and substrate-integrated antimicrobial agents. Surface coatings applied by PVD or sputtering (TiO_2_, TiN, nanosilver films of ~1 µm or less) consistently showed initial antibacterial benefit but faced progressive degradation in the oral environment, with 60% coating loss documented at one month [80] and diminished efficacy from 30 to 60 days [75]. Substrate-integrated approaches (silver infiltrated throughout a tungsten matrix [44], silver nanoparticles incorporated into acrylic [27], silver ions implanted via PIIID [74] maintained antibacterial activity over longer periods because fresh antimicrobial material is continuously exposed as the surface wears. Study design differences also contribute to apparent heterogeneity: the two in situ splint studies suspended oral hygiene during testing, which maximizes biofilm accumulation and may amplify the measurable effect of antibacterial surfaces [74], whereas clinical studies in orthodontic patients with maintained oral hygiene likely reflect more conservative estimates of coating benefit.

The mechanism of antibacterial action varies by coating type, which may explain differential performance against specific organisms. Silver-based coatings act through membrane disruption, respiratory chain inhibition, and DNA replication interference [74]. TiO_2_ and N-doped TiO_2_ rely on photocatalytic generation of reactive oxygen species [75], which requires light activation, a condition partially limited in the dark intraoral environment [77]. One study demonstrated species-dependent susceptibility, with *L. acidophilus* showing less susceptibility to NanoAg-IS-BOA than other cariogenic species [28].

One study that did not evaluate an antibacterial coating found the opposite pattern: Cu-NiTi archwires exhibited greater *S. mutans* adhesion than NiTi wires, attributable to higher surface roughness and surface free energy rather than any antimicrobial benefit from copper content. This finding underscores that alloy composition alone does not guarantee antibacterial performance, and that surface characteristics (roughness, free energy) can dominate adhesion behavior [78].

### 3.6. Synthesis of Results

This section synthesizes findings across all 109 included studies to identify overarching patterns, convergent evidence, knowledge gaps, and translational barriers.

Direct within-study comparisons of in vitro and in vivo performance are available for only a small subset of studies. The QA-GNC aligner coating and AuDAPT aligner coating each incorporated both in vitro bacterial testing and in vivo mucosal safety testing [70], confirming biocompatibility but not providing parallel antibacterial data in both settings. The most important translational data come from studies conducted entirely in vivo or in situ, which reveal a markedly more complex picture than in vitro predictions suggest. Comparisons are shown in Table 3 and Table 4.

The in situ splint studies [44,74] represent the closest available proxy to clinical orthodontic conditions without the confounds of a full treatment context. Silver-infiltrated tungsten matrix material maintained 60.8–78.1% biofilm volume reduction even after simulated 2 years of toothbrushing abrasion [44], and all three silver modification methods (galvanic, PVD, PIIID) significantly reduced biofilm volume and coverage [74]. These in situ effects are quantitatively similar to in vitro data for silver-based coatings, suggesting reasonable translational fidelity for contact-based silver activity.

The in vivo clinical studies provide a markedly different picture for photocatalytic coatings. The N-doped TiO_2_ split-mouth RCT found that while coated brackets significantly reduced *S. mutans* plaque concentrations at both 30 and 60 days, efficacy was better at 30 days than at 60 days, attributed partly to dependence on light activation that diminishes in the oral cavity [75]. The TiO_2_-coated NiTi archwire clinical study documented coating delamination of approximately 60% by 1 month [80], demonstrating that mechanical stability achieved in short-term in vitro tests does not persist under the tribological conditions of archwire sliding in brackets. TiO_2_-coated SS wires evaluated clinically showed consistent antibacterial activity in the mandible over 1–4 weeks without significant decline between weekly measurements [77], contrasting with the mechanical degradation documented for NiTi archwires, likely attributable to different mechanical demands placed on SS versus NiTi wires and whether the archwire is actively sliding.

The two in vivo PMMA appliance studies (Farhadian et al., Ghorbanzadeh et al.) both confirmed clinically meaningful *S. mutans* reductions with silver nanoparticles [27,28]. The Farhadian RCT is notable for its blinding, stratified randomization, and use of ANCOVA to adjust for baseline differences, the strongest clinical evidence in this corpus [27]. The Hashem RCT examining enamel mineral content (Ca and P) in patients with nanosilver-coated brackets documented stable mineral content over 2 months in the coated group and significant decreases in the uncoated group [26], providing the only direct clinical evidence of downstream caries-preventive effects of an antibacterial bracket coating.

Across the 109 included studies, silver-based coatings constitute the most extensively studied category, with reported reductions in bacterial adhesion and biofilm formation ranging from 23% to >99% under controlled laboratory conditions. Silver nanoparticles electrodeposited on NiTi archwires achieved >90% bacterial reduction in vitro [81], smaller AgNPs (8.1 nm) reduced *S. mutans* adhesion on brackets by approximately 98.8% in vitro [82], and one randomized controlled trial examining silver-incorporated removable retainers reported a mean reduction of 40.31 CFU (95% CI 24.83–55.79) in *S. mutans* colony counts at seven weeks [27]. Two in vivo studies using nanosilver-coated brackets, one in rats [79] and one in humans measuring enamel mineral content [26], reported measurable effects under real-world conditions, though the clinical significance of these changes for white spot lesion prevention remains to be established.

TiO_2_-based coatings represent the second most studied category. In vitro studies consistently reported that nitrogen-doped TiO_2_ outperformed undoped TiO_2_ on brackets [12] and archwires [36]. N-doped TiO_2_ achieved a bactericidal rate of 87.2% versus only 5.9% for undoped TiO_2_ on composite archwires under visible light [36], and the N-doped TiO_2_ bracket study maintained stable CFU reduction over 90 days in vitro [12]. However, when TiO_2_ coatings were tested in an actual clinical setting on NiTi archwires, the coating lost approximately 60% of its thickness within one month due to delamination [80], indicating that in vitro durability may not predict intraoral performance. The anatase and rutile phase comparison revealed that rutile TiO_2_ produced greater antibacterial effects but also substantially greater cytotoxicity than anatase [46]. The combination of TiO_2_ with Ag in a single coating (Ag + TiO_2_) produced superior effects versus either material alone in vitro [13], consistent with a synergistic mechanism, though this has not been validated clinically.

ZnO-based coatings demonstrated antibacterial activity across multiple device types in vitro. ZnO NPs applied via CVD on NiTi wires achieved 98.6% reduction in *S. mutans* colonies [39], and ZnO/carbon quantum dot composite bracket coatings achieved 96.13% *S. mutans* reduction under natural light in 24 h [14]. No direct comparison studies, CuO and CuO-ZnO coatings eliminated *S. mutans* colonies entirely within 2 h in vitro [11]. Despite this in vitro activity, no clinical or in vivo validation of ZnO coatings on orthodontic devices has been conducted, representing a critical gap in the evidence base.

Yet, the majority of the available evidence is derived from in vitro studies and should not be interpreted as proof of long-term clinical effectiveness, and the translational gap between preclinical evidence and clinical validation must be highlighted. Among the investigated materials, N-doped TiO_2_ demonstrated a favorable balance of antibacterial activity and reported biocompatibility under laboratory conditions; however, clinical validation remains lacking.

**Table 3:** Antibacterial Efficacy of Coatings by Material Type.

Material	Coating Category	Representative Agents	Bacterial Target	Efficacy Range	No. of Studies	Evidence Level	Key References
**Brackets**	Silver nanoparticles	AgNPs (8–25 nm), Ag-W matrix, Ag-Pt alloy	*S. mutans*	60–99% CFU reduction; ZOI 4.5–10 mm	15	14 in vitro, 1 in situ	Łyczek 2023 [83], Denis 2022 [44], León 2018 [82]
**Brackets**	TiO_2_ photocatalytic	N-doped TiO_2_, anatase/rutile TiO_2_, TiO_2_:Ag	*S. mutans*, *L. acidophilus*	79–98% bacterial reduction; 90–95% antimicrobial rate	12	11 in vitro, 1 RCT	Salehi 2018 [12], Monica 2022 [75], Zhang 2018 [14]
**Brackets**	Metal oxide NPs	ZnO, CuO, CuO-ZnO hybrid	*S. mutans*, *L. acidophilus*	45–96% inhibition; zero colonies at 2 h (CuO)	8	8 in vitro	Zeidan 2022 [10], Sharma 2025 [84], Ramazanzadeh 2015 [11]
**Brackets**	Polymer-based	Polydopamine (PDA), PDA-HCDs, chitosan	*S. mutans*, *E. coli*	78–98% CFU reduction; >80% bacterial killing	6	6 in vitro	Singer 2025 [85], Wang 2023 [86], Mayma 2023 [87]
**Brackets**	Hybrid/composite	Ag-CuO, Ag/ZnO, TiO_2_ + Ag, gold-oxoborate	*S. mutans*, *L. acidophilus*	45–98% reduction; 78% adhesion reduction	6	6 in vitro	Sharma 2025 [84], Fatani 2017 [13], Łyczek 2023 [83]
**Archwires**	Silver nanoparticles	AgNPs (10–50 nm), Ag/PTFE, PDA/PEDOT/AgNP	*S. mutans*, *L. acidophilus*, *S. sanguinis*	50–90% CFU reduction; >90% bacterial reduction	12	12 in vitro	Mhaske 2015 [88], Gil 2020 [81], Lee 2020 [31]
**Archwires**	TiO_2_ photocatalytic	N-doped TiO_2_, TiO_2_:Ag, anatase TiO_2_	*S. mutans*, *P. gingivalis*, *A. actinomycetemcomitans*	74–98% adhesion/biofilm reduction; 87% reduction under visible light	8	7 in vitro, 1 split-mouth	Chun 2007 [89], Liu 2017 [36], Bacela 2022 [29]
**Archwires**	ZnO nanoparticles	ZnO NPs (CVD, sol-gel, precipitation)	*S. mutans*, *S. pyogenes*, *S. aureus*	72–98.6% microbial reduction; complete growth inhibition	7	7 in vitro	Gholami 2021 [39], Kachoei 2016 [35], Hammad 2020 [90]
**Archwires**	Graphene/polymer	Graphene oxide (GO), PDA-GO, lysozyme	*S. mutans*, *S. aureus*	23–93% bacterial adhesion reduction (dose-dependent)	5	5 in vitro	Dai 2022 [91], Chen 2023 [34], He 2020 [92]
**Archwires**	TiN-Cu coating	Titanium nitride with copper	*S. mutans*, *S. mitis*	Significant CFU reduction (*p* < 0.05); sustained 28 days	1	1 in vitro	Ilic 2023 [32]
**Miniscrews**	Silver nanoparticles	AgNPs, Ag/HA NPs, Ag/a-C:H nanocomposite	*S. mutans*, *S. aureus*, *E. coli*	3–6 log reduction; ZOI 10–50 mm^2^	6	6 in vitro	Venugopal 2017 [62], Thukkaram 2020 [64], Abo-Elmahasen 2022 [57]
**Miniscrews**	ZnO-based	ZnO NPs, ZnO-doped TiO_2_ nanotubes + doxycycline	*P. gingivalis*, *S. mutans*, *S. aureus*	ZOI 13–39 mm (Day 5); sustained 30 days with drug	4	4 in vitro	Noorollahian 2022 [58], Mohamed 2025 [63], Othman 2024 [93]
**Miniscrews**	Chitosan-based	Chitosan, chitosan-AgNPs, chitosan + azithromycin	*S. mutans*, *S. sobrinus*, *P. gingivalis*	31–53% biofilm reduction; ZOI 9–40 mm (dose-dependent)	4	4 in vitro	Nguyen 2019 [94], Sreenivasagan 2020 [59], Anggani 2021 [55]
**Aligners/Acrylic**	Gold nanoparticles	QA-GNCs, AuDAPT	*S. mutans*, *P. gingivalis*	55–95% biofilm/viability reduction; growth stopped at 10^4^ CFU/mL	2	2 in vitro	Xie 2020 [70], Zhang 2020 [73]
**Aligners/Acrylic**	ZnO/MgO NPs	ZnO, MgO, ZnO + MgO	*S. mutans*, *Lactobacillus* spp., *P. gingivalis*	Significant CFU reduction; ZOI > 10–25 mm	2	2 in vitro	Gharibnavaz 2025 [95]
**Aligners/Acrylic**	Curcumin-based	Curcumin NPs, Curcumin-Nisin-PLLA	*S. mutans*, *C. albicans*	68–78% reduction; ZOI 3.8–16 mm (dose-dependent)	2	2 in vitro	Soleymanijadidi 2023 [69], Pourhajibagher 2022 [72]
**Aligners/Acrylic**	TiO_2_ photocatalytic	TiO_2_ under UVA	*S. mutans*, *S. sobrinus*, *S. gordonii*	99.9% CFU reduction; viability 0.2–5.4%	1	1 in vitro	Kuroiwa 2018 [68]
**Ligatures**	Silver-based	Nano-Ag fluoride, AgNPs (green synthesis)	*S. mutans*, *S. aureus*, *E. coli*, *L. casei*	57% CFU reduction; 86% biofilm thickness reduction; ZOI present for all species	4	4 in vitro	Choi 2024 [52], Pasala 2024 [96], Schubert 2024 [97], Hernández-Gómora 2017 [98]
**Bands**	Silver/ZnO NPs	Nano-Ag, nano-ZnO	*S. mutans*, *L. acidophilus*, *C. albicans*	2–3.4 log_10_ reduction (Ag); 0.6–2.14 log_10_ (ZnO)	2	2 in vitro	Bahrami 2023 [50], Prabha 2016 [51]

Note: Efficacy Range values are reported in the units employed by each individual study: CFU/mL = CFU per milliliter of suspension; CFU/mm^2^ = CFU per unit surface area; % = percentage reduction from control; log_10_ = base-10 logarithm of CFU reduction; OD = optical density (biofilm biomass); ZOI = zone of inhibition diameter (mm); Ct = cycle threshold (real-time PCR). Direct numerical comparisons across studies require unit harmonization.

**Table 4:** Durability, Biocompatibility, and Mechanical Impact of Selected Coatings.

Coating Type/Material	Durability Finding	Ion Release/Safety Data	Mechanical Impact	Evidence Level	Key References
**Silver NPs on brackets**	Sustained 30 days (electroplated); 2 months (PDA); 3 months (CuO/TiO_2_/HA-SNPs)	Ag ions within safe limits (~2 ppm); no cytotoxicity at therapeutic concentrations	Nano-Ag: no significant friction increase (0.77–0.82 N vs. 0.55 N control)	14 in vitro, 1 in situ	Arash 2016 [42], Singer 2025 [85], Ameli 2022 [43], Ghasemi 2017 [99], Ryu 2012 [47]
**TiO_2_ on brackets**	90 days (N-doped); 3 months storage (nano-TiO_2_); 60% loss after 1 month (clinical)	Grade 0 cytotoxicity; no mucosal irritation; anatase 77–89% viability, rutile 21–40% (severe)	Nano-TiO_2_: significant friction increase (1.52–1.57 N vs. 0.55 N, *p* < 0.05)	11 in vitro, 1 RCT	Salehi 2018 [12], Ghasemi 2017 [99], Venkatesan 2020 [80], Baby 2017 [46], Cao 2016 [100], Cao 2013 [45]
**Ag-W matrix (brackets)**	Maintained after simulated 2-year abrasion	Tungsten considered innocuous; Ag release controlled by matrix	Not assessed	1 in situ	Denis 2022 [44]
**ZnO/CuO on brackets**	28 days (ZnO/CuO NPs); some decline from baseline to 4 months (Ag-CuO hybrid)	Ion release below toxic thresholds at all time points; peak at day 7, declined thereafter	ZnO: 64% friction reduction; chitosan: 53% reduction vs. uncoated	8 in vitro	Mobeen 2022 [48], Sharma 2025 [84], Elhelbawy 2021 [101]
**TiN-Cu on archwires**	Effective 28 days; stable in neutral and acidic environments	Highest cell viability in 28-day eluates; Cu within safety limits; lower Ni release vs. uncoated NiTi	Not assessed	1 in vitro	Ilic 2023 [32]
**AgNPs on archwires**	Stable under orthodontic conditions; up to 42 days (PDA/PEDOT/AgNP system)	Ag^+^ release within safe limits (ICP-MS); good osteoblast morphology; Ni ion release low	Not assessed	12 in vitro	Gil 2020 [81], Lee 2019 [31]
**ZnO on archwires**	Durable after bending and friction cycling; no bacterial growth at 48 h	No viability reduction ≤ 5 µg/mL; 20% reduction at 10 µg/mL; >55% reduction at 25 µg/mL (fibroblasts)	21% friction reduction	7 in vitro	Kachoei 2016 [35]
**TiO_2_:Ag on archwires**	Biofilm reduction declined from 98% (24 h) to 40% (96 h)	Not assessed	Not assessed	7 in vitro, 1 split-mouth	Bacela 2022 [29], Kielan-Grabowska 2021 [102]
**Graphene oxide on archwires**	Stable at 4 weeks under mechanical stress and saliva exposure	Cell viability ~85% after 5 days; meets ISO 10993-5:2009; high GO induces oxidative stress	Not assessed	5 in vitro	Chen 2023 [34], Dai 2021 [91]
**ZnO-doped TiO_2_ nanotubes + doxycycline (Miniscrews)**	Sustained antimicrobial action 30 days; progressive decline in ZOI from Day 5 to Day 30	Not assessed	Not assessed	4 in vitro	Noorollahian 2022 [58]
**QA-GNCs on aligners**	Maintained effectiveness > 3 months and after > 3 usage cycles	Negligible toxicity in vitro; no inflammatory response or mucosa irritation in vivo; safe up to 4.22 µg/cm^2^	Not assessed	2 in vitro (1 with in vivo biocompatibility)	Xie 2020 [70]
**Curcumin NPs in acrylic**	Sustained release ≥ 30 days (curcumin NPs); significant at 30 d, diminishing by 60 d (Cur-Nis-PLLA)	All tested concentrations (0.5–5%) non-cytotoxic	All groups > 50 MPa flexural strength; 5% acceptable, 10% unacceptable (Cur-Nis-PLLA)	2 in vitro	Soleymanijadidi 2023 [69], Pourhajibagher 2022 [72]

## 4. Discussion

### 4.1. Scoping Review Methodology and Rationale

This review employed a scoping review methodology rather than a traditional systematic review with meta-analysis for several critical reasons that warrant explicit discussion, as the choice of review type fundamentally shapes the interpretation and application of findings. The 109 included studies span in vitro materials science experiments (88%), animal models (5%), and clinical trials (7%), with no standardized protocols for coating application, bacterial challenge, or outcome measurement. In vitro studies alone encompass diverse bacterial models (single-species reference strains, multi-species biofilms, clinical isolates), outcome measures (CFU enumeration, zone of inhibition, biofilm biomass, viability staining, microscopy, molecular assays), exposure durations (hours to weeks), and coating characterization methods (SEM, EDS, XPS, XRD, ion release kinetics). This methodological diversity precludes meaningful meta-analysis, as pooling effect sizes across heterogeneous assays would produce misleading estimates that obscure rather than clarify the evidence [18].

Formal risk-of-bias tools designed for clinical trials (e.g., Cochrane Risk of Bias 2.0, ROBINS-I) or animal studies (e.g., SYRCLE’s RoB tool) assess domains such as randomization, allocation concealment, blinding, and selective outcome reporting—constructs that are not applicable to in vitro materials characterization studies, which constitute most of the evidence base. While quality considerations for laboratory research exist (e.g., use of validated bacterial strains, adequate replication, appropriate statistical analysis, transparent reporting), no consensus framework has been established for formal bias assessment in materials science research. Applying clinical trial bias assessment tools to in vitro studies would be methodologically inappropriate and would not yield interpretable or actionable quality scores. Instead, we systematically extracted and reported key methodological characteristics (bacterial model, outcome measure, coating characterization, durability assessment) to enable readers to contextualize findings and assess translational relevance.

Antibacterial coatings for orthodontic appliances remain an emerging technology without established clinical standards, regulatory approval pathways, or consensus on optimal materials and mechanisms. The objective of this review was to map the breadth of approaches, compare reported performance across coating categories, characterize the state of preclinical and clinical evidence, and identify candidates and knowledge gaps for further translational evaluation [19]. Scoping reviews are explicitly designed for fields where the evidence is heterogeneous, the research questions are broad, and the goal is to inform future research directions rather than to provide definitive answers to narrow clinical questions [20].

No studies reported clinical endpoints relevant to orthodontic practice (white spot lesion incidence, periodontal indices over treatment duration). The remaining clinical and in vivo studies measured surrogate outcomes (microbial counts) over short durations (1–12 weeks), which are insufficient to assess clinically meaningful protection over full orthodontic treatment (18–24 months). Meta-analysis of such heterogeneous short-term surrogate outcomes would produce pooled estimates that lack clinical interpretability and could mislead clinicians regarding real-world efficacy. By employing a scoping review approach, we transparently report the diversity of outcomes and durations without artificially aggregating incomparable data.

By mapping the evidence landscape, this scoping review identifies critical knowledge gaps and methodological inconsistencies that must be addressed before definitive efficacy evaluations can be conducted. Specifically, future research requires:

1. Standardized in vitro testing protocols (bacterial strains, biofilm maturation time, outcome measures, statistical analysis) to enable cross-study comparison and reproducibility.
2. Long-term intraoral durability studies (≥12 months) under realistic mechanical and chemical stress to assess coating stability over full treatment duration.
3. Adequately powered, multi-center randomized clinical trials with white spot lesion incidence as the primary endpoint and follow-up extending through debonding and retention.
4. Post-market surveillance for rare adverse events (e.g., argyria, hypersensitivity reactions, systemic absorption of nanoparticles).

Once this foundational work is completed, systematic reviews with meta-analysis and formal certainty-of-evidence assessment (e.g., GRADE) will become feasible and will provide the definitive evidence needed for clinical practice guidelines.

Readers should recognize that this review does not provide pooled effect estimates, does not formally assess risk of bias using validated tools, and does not grade certainty of evidence using GRADE or similar frameworks. The narrative synthesis and reported ranges reflect the diversity of findings across heterogeneous protocols, not confidence intervals around a true effect size. Clinical recommendations are intentionally framed as hypothesis-generating rather than practice-changing. Clinicians should not adopt antibacterial coatings for routine use based on the current evidence base, which remains predominantly preclinical. Instead, the field requires the standardized protocols and long-term clinical trials identified above before evidence-based clinical recommendations can be made.

While scoping reviews do not apply formal bias assessment or certainty grading, they are not methodologically inferior to systematic reviews; rather, they serve a different and complementary purpose [5,22]. Scoping reviews are appropriate when the goal is to map an emerging field, identify research gaps, and inform future study design, precisely the objectives of this review. We have followed established scoping review guidelines (PRISMA-ScR, Arksey and O’Malley framework, Levac refinements) and have transparently reported our methods, findings, and limitations. The scoping approach enables us to provide a comprehensive evidence map that would not be possible with a narrowly focused systematic review, while avoiding the misleading precision of meta-analyses applied to heterogeneous data.

In summary, the scoping review methodology was deliberately chosen to match the exploratory objectives and heterogeneous evidence base of this field. This review provides the foundation necessary for future studies by identifying evidence based antibacterial coatings (silver-based, nitrogen-doped TiO_2_), highlighting critical knowledge gaps (durability, long-term clinical endpoints), and establishing the need for standardized protocols. The findings should be interpreted as hypothesis-generating and as a call for rigorous translational research, not as definitive evidence for clinical implementation.

Across the 109 studies included in this review, antibacterial surface coatings consistently reduced bacterial adhesion and biofilm formation on orthodontic appliance surfaces compared to uncoated controls. This directional consistency is robust and reproducible across coating types, device substrates, and research groups. However, the strength of this conclusion must be qualified by the evidence hierarchy: 88% of included studies are purely in vitro, most conducted over periods of hours to days under static, monospecies conditions that do not replicate the mechanical loading, salivary flow, and polymicrobial complexity of the oral environment. The magnitude and clinical durability of the antibacterial effect under real treatment conditions remain substantially uncertain.

### 4.2. In Vivo and Clinical Evidence

The 13 in vivo and clinical studies identified provide limited but directionally supportive evidence. Reported reductions ranged from approximately 50% for surface-deposited TiN coatings on stainless steel wires (*p* = 0.03) [76] to over 90% for silver nanoparticle formulations in PMMA appliance baseplates [88]. The largest blinded RCT (n = 61) found a mean difference of 40.31 *S. mutans* CFU (95% CI: 24.83–55.79; *p* < 0.001) favoring silver nanoparticle-containing retainers at seven weeks [27], and a silver-infiltrated tungsten bracket material achieved 60–78% biofilm volume reduction persisting after simulated two-year abrasion [44]. No clinically significant adverse effects were reported across any coating type, though long-term systemic exposure data remain absent [27]. These results are encouraging but must be interpreted cautiously: follow-up periods across clinical studies ranged from two to twelve weeks, none approached the 18–24 months of a full orthodontic treatment course, and no study reported clinical endpoints such as WSL incidence or periodontal attachment loss. The biological relevance of short-term CFU reductions for long-term caries prevention therefore cannot be established from the available evidence.

### 4.3. Durability as the Principal Determinant of Clinical Relevance

The principal source of heterogeneity across studies is coating durability rather than the presence or absence of an antibacterial effect per se. Thin-film coatings applied by physical vapor deposition (PVD) or sputtering, particularly TiO_2_ on archwires, showed substantial degradation in the oral environment, with approximately 60% coating loss at one month [80] and declining efficacy between 30 and 60 days for N-doped TiO_2_ brackets [75]. In contrast, substrate-integrated approaches, silver nanoparticles incorporated throughout acrylic bulk [27], silver vacuum-infiltrated into tungsten matrices [44], and plasma ion immersion implantation (PIIID) [74], maintained antibacterial activity over clinically relevant timeframes. These findings suggest that coating architecture is a more important determinant of sustained efficacy than coating material alone: bulk-incorporated or structurally integrated systems consistently outperform surface-deposited films under intraoral mechanical and chemical challenge.

### 4.4. The In Vitro to In Vivo Translational Gap

The discordance between in vitro and clinical performance is most clearly illustrated by TiO_2_ nanoparticle coatings on NiTi archwires, which demonstrated robust antibacterial activity in vitro yet lost approximately 60% of their coating thickness within one month of clinical use due to mechanical delamination under archwire-bracket sliding forces [80]. This failure mode is structurally predictable: NiTi archwires undergo continuous flexion in the bracket slot, a dynamic loading condition entirely absent from static in vitro incubation. By contrast, silver coatings on stainless steel wires, which experience substantially less cyclic deformation, maintained their antibacterial effect over four weeks clinically [77], and silver-containing PMMA baseplates performed consistently across in vitro and clinical settings [27], reflecting the low-deformation environment of removable appliances. This device-specific mechanical context explains why translational fidelity appears higher for brackets and appliance bases than for archwires and underscores the need for in vitro models that incorporate mechanically realistic loading conditions before clinical testing is warranted.

A second dimension of this translational gap is biological complexity. The large majority of in vitro antibacterial assessments in the included studies were conducted using single-species planktonic cultures or early-stage biofilms, organisms that are operationally convenient but represent poor surrogates for the polymicrobial community that colonises orthodontic surfaces in vivo. In the oral cavity, orthodontic appliances acquire a salivary pellicle that conditions subsequent microbial adhesion, and the resulting biofilm community is repeatedly challenged by environmental stressors absent from standard in vitro protocols: sucrose pulses that drive cyclical pH excursions below the critical demineralisation threshold, continuous salivary flow that dilutes antimicrobial agents and renews the pellicle, masticatory and archwire-bracket sliding shear, and repeated mechanical disruption from brushing and interdental hygiene procedures. The few studies in this review that employed salivary-inoculum or multispecies biofilm models consistently reported lower antibacterial efficacy than single-species counterparts, indicating that the percentage reductions derived from simplified in vitro systems likely overestimate the antimicrobial protection achievable under realistic intraoral conditions.

### 4.5. Light Dependence of Photocatalytic Coatings

TiO_2_-based and related photocatalytic coatings achieve their antibacterial effect through reactive oxygen species generated under light activation, a mechanism that is well-established in vitro but constrained in the largely dark oral cavity. Nitrogen-doped TiO_2_ was developed specifically to shift the activation spectrum from UV to visible light [75], and the corresponding clinical RCT confirmed efficacy at 30 days; however, the effect declined by 60 days [75], consistent with limited sustained light activation under clinical conditions. Cobalt-doped zinc ferrite (CZFO), requiring only halogen light rather than UV-A [103], may represent a more practically viable photocatalytic approach, though it has been evaluated in only a single bracket study. For clinical contexts where light activation cannot be reliably assured, contact-killing mechanisms, quaternary ammonium compounds, gold nanoclusters, chitosan, offer a more mechanistically appropriate strategy.

### 4.6. Dose-Response Relationships and Biocompatibility

Several studies quantified concentration-response relationships relevant to formulation optimization. Polydopamine-graphene oxide showed a clear dose-dependent increase from 33.4% (lowest GO loading) to 93.1% (highest GO loading) [34] mirroring concentration-dependent cytotoxicity in the same system. The Ag/a-C:H nanocomposite showed superior antibacterial performance at higher silver current densities while maintaining ≥90% cell viability across tested concentrations [64]. Most coatings demonstrate acceptable cytotoxicity at orthodontically relevant concentrations; however, the rutile phase of TiO_2_ produces moderate-to-severe cytotoxicity relative to anatase effects [46], this should be interpreted with caution, as it derives from a single in vitro study and TiO_2_ cytotoxicity is highly dependent on particle size, morphology, surface chemistry, dose, agglomeration state, illumination, exposure duration, and cell type, higher silver concentrations (>3 wt%) provided only little additional antibacterial benefit while raising concerns regarding cytotoxicity and tissue staining [48,79]. These findings confirm that optimal formulation requires identifying the concentration window at which antibacterial efficacy is maximized without exceeding cytotoxicity thresholds, a balance that has not been systematically evaluated under clinically realistic exposure conditions for most coating systems.

In the broader dental biomaterials context, a recent study by Jamali et al. shows that plasma electrolytic oxidized (PEO) TiO_2_ systems, ternary co-incorporation of calcium, phosphorus, and copper into the oxide layer illustrates how multi-elemental formulation can simultaneously optimize this balance: the Cu-containing ternary coating significantly restored bactericidal activity against *Escherichia coli* relative to binary (Ca + P) and unmodified PEO layers, while MG-63 osteoblastic cell integration, proliferation, and viability were considerably improved rather than impaired, demonstrating that Cu at the concentrations achieved through PEO did not compromise biocompatibility [104]. Critically, this formulation also enhanced corrosion potential by 187 mV and reduced corrosion current density by one order of magnitude in artificial saliva solution [104], underscoring that biocorrosion stability, a dimension rarely integrated into dose-response evaluations, is itself concentration-dependent and must be considered alongside antibacterial and cytotoxic endpoints. These findings confirm that optimal formulation requires identifying the concentration window at which antibacterial efficacy is maximized without exceeding cytotoxicity thresholds. This balance has not been systematically evaluated under clinically realistic exposure conditions for most coating systems, but this multi-element surface modification principle increasingly relevant to TiO_2_-based strategies on titanium orthodontic substrates such as miniscrews and archwires [104].

### 4.7. Comparative Evidence by Coating Type

The evidence base for each coating category can be stratified by translational level as follows.

Clinical and in vivo evidence (highest translational relevance): Silver-incorporated PMMA appliance baseplates have the strongest clinical support, with *S. mutans* reductions confirmed in a double-blind RCT (n = 61) over 4–7 weeks [27]. N-doped TiO_2_ brackets demonstrated efficacy in one split-mouth RCT at 30 days, though the effect diminished by 60 days [75].

In vitro evidence (preclinical, proof-of-concept only): Among in vitro studies, hybrid and combination coatings (Ag/ZnO > Ag > ZnO [10]; CuO-ZnO > CuO > ZnO [11]; N-doped TiO_2_ > TiO_2_ [36]; TiO_2_:Ag > TiO_2_ [29]; Ag+ TiO_2_ > Ag > TiO_2_ [13] systematically outperformed single-material counterparts across multiple independent research groups. Polymer-based coatings (chitosan, polydopamine, PEG, curcumin) offer consistently favorable biocompatibility profiles but generally lower antibacterial magnitude in vitro; their primary role may be as carrier matrices or surface functionalization layers [29,37,60,87,94]. These in vitro findings require in vivo validation before clinical implications can be drawn.

The negative finding for TiN and calcium phosphate-doped TiN bracket coatings, no statistically significant biofilm reduction (*p* = 0.06) [49], contrasts with the positive in vivo result for TiN-coated stainless steel wires [76]. This discrepancy likely reflects differences in deposition method (cathodic cage deposition versus PVD), device geometry, and outcome assay (crystal violet at 24 h versus clinical CFU sampling), illustrating that coating materials cannot be evaluated independently of their deposition method and device context.

### 4.8. Sterilization Compatibility

The single study examining sterilization effects on ZnO NP-coated miniscrews found that autoclave sterilization substantially eliminated antibacterial activity against *S. mutans* while partially preserving it against *S. aureus* [63]. This finding carries direct clinical relevance: miniscrews are sterilized prior to insertion, meaning sterilization-sensitive coatings must either be applied post-sterilization or demonstrate resistance to the sterilization process, a criterion that most reviewed coatings have not been evaluated against.

### 4.9. Methodological Limitations of the Included Studies

Beyond the in vitro predominance, several methodological weaknesses limit the interpretability of the evidence base. First, bacterial strain selection varied widely across studies, with most using single-species monocultures of *S. mutans* or *S. aureus* that do not reflect the polymicrobial biofilm ecology of the orthodontic oral environment; the few studies using multispecies or salivary inoculum models consistently reported lower antibacterial efficacy than monospecies counterparts, suggesting that published reduction percentages may overestimate real-world performance. This overestimation reflects environmental stressors that standard in vitro protocols fail to replicate: orthodontic appliances acquire a salivary pellicle that conditions biofilm adhesion and partially shields bacteria from surface-released antimicrobials; sucrose pulses repeatedly lower local pH below the demineralisation threshold, cycling the ionic environment around the coating surface; salivary flow continuously dilutes agents and renews the pellicle layer; and masticatory and archwire-bracket sliding shear, together with periodic mechanical disruption from brushing and interdental hygiene procedures, impose forces on biofilm stability that static immersion assays cannot reproduce. Second, outcome heterogeneity was substantial: CFU counting, crystal violet assay, live/dead fluorescence microscopy, and biofilm mass quantification are not interchangeable, yet are frequently compared without assay sensitivity or biological relevance. Third, exposure durations in vitro ranged from 2 h to 90 days, making cross-study comparisons of durability unreliable. Fourth, no included study reported WSL incidence, caries increment, or periodontal attachment change as a primary outcome, the clinical endpoints most relevant to the stated purpose of these coatings.

### 4.10. Future Research Priorities

Advancing this field toward clinical translation requires addressing several specific methodological and translational gaps. First, standardized in vitro testing protocols are needed that incorporate multispecies biofilms, mechanically realistic loading conditions (particularly for archwires), and clinically relevant exposure durations (≥12 weeks) to enable meaningful cross-study comparisons and improve the predictive validity of preclinical models. Second, in vivo studies must extend beyond the current 8–12 week timeframes to evaluate coating performance over treatment-relevant durations (12–24 months for fixed appliances). Third, adequately powered, randomized, double-blind clinical trials are required with white spot lesion incidence or caries increment, rather than CFU counts alone, as primary endpoints, since short-term bacterial load reduction has not been shown to predict clinically meaningful caries prevention in the orthodontic context. Fourth, systematic evaluation of sterilization compatibility is essential for all coatings intended for fixed appliances or miniscrews, as the single study examining this question found that autoclave sterilization substantially eliminated antibacterial activity for ZnO coatings. Fifth, long-term intraoral durability must be rigorously assessed, as the available clinical evidence indicates that surface-deposited coatings may degrade substantially within 1–3 months, potentially limiting their protective effect over full treatment courses. Finally, the regulatory pathway and manufacturing scalability of candidate coating systems must be assessed before clinical trial initiation can be justified.

Based on the current evidence base, silver-based coatings (particularly AgNP-incorporated PMMA for removable appliances and electrodeposited or composite AgNP systems for brackets) and nitrogen-doped TiO_2_ coatings warrant prioritization in future clinical trial pipelines due to their combination of in vitro efficacy, preliminary clinical data, and acceptable biocompatibility profiles. However, clinicians should await the results of adequately powered randomized controlled trials with clinically relevant endpoints before adopting any antibacterial coating system for routine clinical use.

## 5. Conclusions

This scoping review maps a heterogeneous evidence base of 109 studies, the large majority of which were conducted in vitro. Antibacterial surface coatings demonstrated the potential to reduce bacterial adhesion and biofilm formation under controlled laboratory conditions. Silver-containing formulations, nitrogen-doped TiO_2_, TiN-Cu, and Ag/ZnO systems showed favorable laboratory profiles and may warrant further investigation; however, the comparative evidence is heterogeneous, clinical studies are few and short-term, and no coating has demonstrated prevention of white spot lesions or caries over a full orthodontic treatment course. Current evidence therefore supports antibacterial potential rather than routine clinical implementation. Standardized multispecies biofilm models, realistic mechanical and chemical aging, long-term safety assessment, and adequately powered randomized clinical trials with clinically meaningful endpoints are required before clinical recommendations can be made.

This scoping review maps a heterogeneous evidence base of 109 studies. Antibacterial surface modifications and coating strategies for orthodontic appliances demonstrated substantial reduction in bacterial colonization and biofilm formation under controlled laboratory conditions, the current evidence base remains predominantly preclinical. Silver-containing formulations, nitrogen-doped TiO_2_, TiN-Cu, and Ag/ZnO combination coatings warrant prioritization in future clinical trial pipelines based on their balance of in vitro efficacy, preliminary clinical data, and acceptable biocompatibility. However, rigorous clinical validation is essential before these technologies can be adopted for routine clinical use pending results of adequately powered randomized controlled trials.

While antibacterial surface coatings show considerable results for reducing bacterial colonization on orthodontic appliances, the current evidence is predominantly based on in vitro studies. Therefore, well-designed in vivo studies and long-term clinical trials are required to validate their clinical effectiveness, durability, and safety before widespread clinical implementation can be recommended. Antibacterial coatings remain predominantly preclinical technologies. Clinicians should not adopt these coatings for routine use based on current evidence. Instead, the field requires standardized in vitro testing protocols, long-term intraoral durability studies (≥12 months), and adequately powered randomized clinical trials with white spot lesion incidence as the primary endpoint. This scoping review provides the evidence map necessary to design these future studies. Silver-based and nitrogen-doped TiO_2_ coatings warrant prioritization in clinical trial pipelines, but the null hypothesis, that antibacterial coatings do not provide clinically meaningful protection against white spot lesions during orthodontic treatment, cannot yet be rejected based on the available evidence.

## Figures and Tables

**Table 1:** Summary of Included Studies by Orthodontic Material.

Orthodontic Material	No. of Studies (In Vitro/In Vivo/Clinical)	Dominant Coating Categories	Bacterial Species Most Frequently Tested	Primary Outcome Measures
Brackets	37 (36/1/0)	Silver-based (15), TiO_2_-based (12), Metal oxide NPs (8), Hybrid coatings (6)	*S. mutans* (35), *L. acidophilus* (8), *C. albicans* (4)	CFU counts, zone of inhibition, biofilm biomass/thickness
Archwires	34 (34/0/0)	Silver-based (12), TiO_2_-based (8), ZnO-based (7), Polymer/graphene (5)	*S. mutans* (28), *S. aureus* (8), *E. coli* (6)	CFU counts, bacterial adhesion, biofilm formation, weight increase
Miniscrews	14 (14/0/0)	Silver-based (6), ZnO-based (4), Chitosan-based (4), TiO_2_-based (2)	*S. mutans* (10), *S. aureus* (8), *E. coli* (5), *P. gingivalis* (4)	Zone of inhibition, CFU counts, biofilm inhibition
Aligners/Acrylic	10 (10/0/0)	Gold NPs (2), ZnO/MgO (2), Curcumin-based (2), TiO_2_ photocatalytic (1), Silver-loaded (2)	*S. mutans* (8), *C. albicans* (3), *P. gingivalis* (2)	Biofilm biomass, CFU counts, zone of inhibition, cell viability
Ligatures	5 (5/0/0)	Silver-based (4), Chlorhexidine (1)	*S. mutans* (5), *S. aureus* (2), *E. coli* (2)	Zone of inhibition, CFU reduction, biofilm thickness
Bands	2 (2/0/0)	Silver NPs (2), ZnO NPs (1)	*S. mutans* (2), *L. acidophilus* (1), *C. albicans* (1)	Log_10_ CFU reduction, zone of inhibition
In Vivo/Clinical	13 (0/8/5)	Silver-based (6), TiO_2_-based (4), TiN (1), PEG (1), Cu-NiTi alloy (1), Gold NPs (1)	*S. mutans* (10), Total biofilm (3), *L. salivarius* (2), *P. gingivalis* (2)	CFU counts, real-time PCR (Ct values), biofilm volume (CLSM), enamel demineralization

**Table 2:** In Vivo and Clinical Studies.

Author, Year	Study Design	Material & Coating	Bacterial Outcome/Measure	Coated Result	Control Result	Duration	Key Finding
**Meyer-Kobbe 2018** [74]	In situ (occlusal splint), randomized	SS bracket material; Ag via galvanic, PVD, PIIID	Total biofilm (CLSM)	43–57% dead/live ratio	Significantly lower than control	48 h	Significant biofilm reduction with silver coatings
**Monica & Padmanabhan 2022** [75]	Split-mouth RCT	SS brackets; N-doped TiO_2_ (RF magnetron sputtering)	*S. mutans* (real-time PCR, Ct value)	Ct 38.54 at 30 d; reduced efficacy at 60 d	Ct 34.71 at 30 d	60 days	Reduced *S. mutans* at 30 d (*p* = 0.005), but diminished by 60 d
**Amini et al. 2017** [76]	Split-mouth	SS wires; TiN via PVD	Total bacterial CFU (blood agar)	4 ± 3.4 × 10^4^ CFU	8 ± 7.4 × 10^4^ CFU	4 weeks	~50% CFU reduction (*p* = 0.03)
**Mollabashi et al. 2020** [77]	Split-mouth, block randomized	SS wires; TiO_2_ via PVD (100 nm)	*S. mutans* (CFU counting)	Lower *S. mutans* in weeks 1 and 3	Variable by jaw	4 weeks	Lower *S. mutans* counts, but effect variable by jaw location
**Farhadian 2016** [27]	Parallel-group RCT	Acrylic retainer baseplates; AgNPs (40 nm, 500 ppm)	*S. mutans* (CFU counting)	Mean difference 40.31 CFU favoring coated	Control higher	7 weeks	Significant *S. mutans* reduction (95% CI: 24.83–55.79, *p* < 0.001)
**Denis 2022** [44]	In situ (occlusal splint), randomized	Tungsten bracket material; Ag vacuum-infiltrated	Total biofilm (CLSM)	60–78% biofilm volume reduction	Maintained after 2-year abrasion simulation	2 years (simulated)	Sustained biofilm reduction after simulated 2-year wear
**Ghorbanzadeh et al. 2015** [28]	Double-blind crossover RCT	Orthodontic appliance baseplates; AgNPs (0.5% *w*/*w*) in PMMA	*S. mutans*, *S. sobrinus*, *L. acidophilus*, *L. casei* (CFU)	NanoAg-IS: 70.2–98.4% reduction; NanoAg-I: 30.9–86.1%	Control higher	4 weeks	Significant planktonic bacteria reduction (*p* < 0.05)
**Abraham 2017** [78]	Crossover	Cu-NiTi vs. NiTi archwires	*S. mutans* (real-time PCR)	Cu-NiTi showed *greater* adhesion than NiTi	NiTi lower adhesion	Not specified	Cu-NiTi exhibited increased *S. mutans* adhesion (negative result)
**Rodriguez-Fernandez 2022** [61]	In vitro (included for comparison)	Ti miniscrews; PEG via plasma polymerization	*S. sanguinis*, *L. salivarius* (CFU/mm^2^)	*S. sanguinis*: 300 CFU/mm^2^; *L. salivarius*: 900 CFU/mm^2^	*S. sanguinis*: 600 CFU/mm^2^; *L. salivarius*: 10,000 CFU/mm^2^	2 h	50% reduction (*S. sanguinis*), 90% (*L. salivarius*)
**Zhang 2020** [73]	In vitro + in vivo (animal biocompatibility)	Invisalign aligners; AuDAPT gold NPs (<4 nm)	*P. gingivalis* (OD, SEM, CFU)	0.04 ± 0.01 biofilm OD; growth inhibited at 10^4^ CFU/mL	0.09 ± 0.02 biofilm OD	In vitro only	~55% biofilm reduction; excellent in vivo biocompatibility
**Metin-Gürsoy 2017** [30]	Controlled animal study	SS brackets; Nanosilver via PVD (1 µm)	*S. mutans* (CFU/mL, microscopy)	Significant *S. mutans* reduction	Control higher	75 days	Significant reduction in *S. mutans* CFU/mL over 75 days
**Hashem et al. 2022** [26]	RCT	SS brackets; AgNPs (~25 nm) via chemical bath	Enamel demineralization (SEM, EDX)	Enamel protection maintained	Control showed demineralization	1–2 months	Enamel protection over 2 months
**Metin-Gürsoy et al. 2016** [79]	In vivo animal study (biocompatibility)	SS brackets; Nanosilver via PVD (1 µm)	Tissue histology (inflammatory response)	Similar inflammatory response to standard brackets	Standard brackets	60 days	No increased inflammation vs. standard brackets
**Venkatesan 2020** [80]	Prospective split-mouth clinical study	NiTi archwires; TiO_2_ NPs via RF magnetron sputtering (~81 nm)	*S. mutans* (real-time PCR, Ct value)	Ct 37.00 at 30 d; 60% coating loss after 1 month	Ct 30.97 at 30 d	30 days	Higher Ct (lower adhesion) at 30 d (*p* = 0.0005), but rapid coating degradation

## Data Availability

No new data were created or analyzed in this study. Data sharing is not applicable to this article.

## Supplementary Materials

The following supporting information can be downloaded at: https://www.mdpi.com/article/10.3390/ma19173644/s1, Supplementary File S1: PRISMA-ScR Checklist; Supplementary File S2: Materials and Methods and Results; Supplementary File S3: Table S2 Complete Search Strategies; Supplementary File S4. Methodological characteristics extracted.
