# Supplementary material for: Antibacterial Interventions for Orthodontic Appliances; Surface Modifications, Coatings and Bulk-Incorporated Antibacterial Agents: Materials, Mechanisms and Clinical Application—A Scoping Review
Source: Materials (Basel). 2026 Aug 27;19(17):3644. doi: 10.3390/ma19173644 (PMC13566774; doi:10.3390/ma19173644)
Supplement: Supplementary file 1 [file materials-19-03644-s001.zip › Supplementary File S2. Materials and Methods and Results.pdf]

Biological Relevance Hierarchy and Evidence Classification Framework

To enable systematic comparison of findings across studies with markedly different experimental designs, a four-tier biological relevance hierarchy was applied to classify the translational strength of each included study. This framework, adapted from frameworks proposed for preclinical antimicrobial research that classifies studies based on four domains that collectively determine how closely the experimental conditions approximate the clinical orthodontic environment: (1) biological model complexity, (2) experimental duration, (3) mechanical realism, and (4) outcome measure clinical relevance. Each domain is assigned a level from 1 (lowest translational relevance) to 4 (highest translational relevance), and the overall tier is determined by the modal or minimum level across domains.

Table A. Biological Relevance Hierarchy for Antibacterial Coating Studies in Orthodontics

Tier	Label	Biological Model	Duration	Mechanical Realism	Outcome Measure	Translational Interpretation
Tier 1	Proof-of-Concept	Single-species planktonic suspension (reference strain)	<24 h	None (static immersion)	ZOI diameter or CFU at single time point	Demonstrates material has intrinsic antibacterial activity; no predictive value for clinical performance
Tier 2	Mechanistic Evidence	Single-species biofilm (24–72 h maturation; reference or clinical strain)	24–96 h	None or minimal (artificial saliva)	CFU enumeration or biofilm biomass with multiple time points and replicates	Demonstrates coating can inhibit biofilm formation under controlled conditions; limited translational relevance
Tier 3	Translational Evidence	Multi-species biofilm or salivary inoculum; coating durability assessed post-aging (thermocycling, mechanical abrasion)	≥2 weeks	Simulated mechanical loading or pH cycling	Multiple outcome measures; ion release kinetics; biocompatibility assessed	Provides evidence relevant to intraoral performance; still preclinical; supports clinical trial design

Tier 4	Clinical Validation	In vivo animal model or human clinical trial; randomized design preferred	≥4 weeks	Actual intraoral conditions	Clinical endpoints (WSL incidence, periodontal indices, microbial counts in vivo) or validated surrogate endpoints	Direct evidence of clinical performance; highest translational relevance

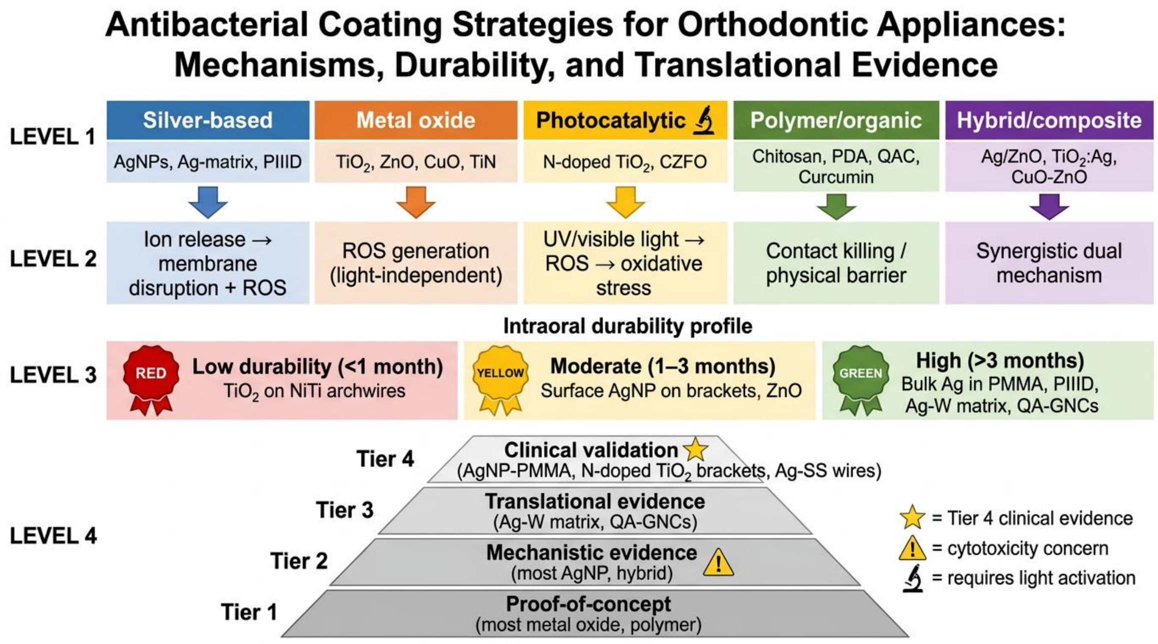

Conceptual framework of antibacterial coating strategies for orthodontic appliances: mechanisms of action, durability profiles, and translational evidence levels. Conceptual overview of antibacterial coating strategies evaluated for orthodontic appliances. The framework integrates coating category, primary antibacterial mechanism, intraoral durability profile (color-coded: green = >3 months; yellow = 1–3 months; red = <1 month), and translational evidence tier (Tier 1–4, as defined in Section 2.8). Coatings marked with ★ have Tier 4 (clinical or in vivo) validation. Coatings marked with ⚠ exhibit concentration-dependent cytotoxicity at or near therapeutically relevant concentrations. Coatings marked with 🔬 require exogenous light activation for antibacterial activity. PIIID = plasma immersion ion implantation and deposition; QAC = quaternary ammonium compounds; QA-GNCs = quaternary ammonium gold nanoclusters; PDA = polydopamine; CZFO = cobalt-doped zinc ferrite; ROS = reactive oxygen species.

Eligibility Criteria

1. Inclusion Criteria
Studies were included if they met all the following criteria:
Study design: Original research articles including in vitro laboratory studies, in vivo animal studies, and clinical trials (randomized controlled trials, non-randomized controlled trials, cohort studies, and case-control studies).

Population: Any orthodontic appliance or device intended for intraoral use, including but not limited to stainless steel brackets, ceramic brackets, archwires (stainless steel, nickel-titanium, beta-titanium), elastomeric ligatures, clear aligners, orthodontic mini-screws, and acrylic removable appliances.

Intervention: Application of antibacterial coatings, surface modifications, or incorporation of antibacterial agents (true thin-film coatings; plasma/ion-implantation surface treatments; and bulk-incorporated antibacterial agents in appliance matrix materials, with exclusion of adhesive/cement studies) to the surface or bulk of orthodontic materials. For the purposes of this review, the following intervention categories are considered within scope: surface-deposited thin-film coatings applied by physical vapour deposition (PVD), chemical vapour deposition (CVD), electrodeposition, sol-gel, dip coating, or sputtering; plasma-based and ion-implantation surface treatments, including plasma polymerization and plasma immersion ion implantation and deposition (PIIID), which modify surface chemistry at the nanoscale without a discrete deposited layer; and bulk-incorporated antibacterial agents distributed throughout the appliance matrix material (e.g., AgNPs or quaternary ammonium compounds in PMMA acrylic resin, silver-infiltrated tungsten alloy). Copper-containing NiTi alloys (Cu-NiTi) were included because their antibacterial activity arises from surface-released copper ions, functionally equivalent to a metal-based surface modification. Studies focused exclusively on antibacterial additives in orthodontic adhesives, cements, or bonding resins were excluded.

Comparison: Presence of a control group consisting of uncoated, unmodified, or conventionally treated orthodontic materials.

Outcome measures: Quantitative or qualitative assessment of antibacterial activity through at least one of the following: bacterial adhesion assays, biofilm formation assays, colony-forming unit (CFU) counts, zone of inhibition measurements, scanning electron microscopy (SEM) imaging of bacterial colonization, live/dead bacterial staining, clinical assessment of white spot lesions, plaque index, gingival index, or periodontal indices.

Language: Articles published in English.

Publication period: No date restrictions were applied to maximize comprehensiveness.

2.. Exclusion Criteria
Studies were excluded if they met any of the following criteria:

Study type: Literature reviews, systematic reviews, meta-analyses, narrative reviews, editorials, commentaries, conference abstracts without full text, case reports, and case series.

Intervention scope: Studies investigating antibacterial properties of orthodontic bonding adhesives, cements, or elastomeric chains without surface coating of the appliance itself; studies on dental implants not used for orthodontic anchorage; studies on general dental materials without orthodontic application.

Lack of control group: Studies without an appropriate uncoated or unmodified control group for comparison.

Outcome measures: Studies reporting only mechanical properties, corrosion resistance, or biocompatibility without antibacterial assessment.
Duplicate publications: When multiple publications reported the same data from the same study population, only the most complete or recent publication was included.

Study Selection Process

1. Screening Phases
Study selection was conducted in three phases:

Phase 1: Deduplication

All retrieved records were imported into Covidence, which automatically identified and removed duplicate records based on title, author, and publication year. Additional manual deduplication was performed to identify near-duplicates with minor variations in bibliographic data.

Phase 2: Title and Abstract Screening

Two reviewers (B.F.-A. and D.N.-J.) independently screened the titles and abstracts of all unique records against the predefined eligibility criteria. Studies were categorized as "include," "exclude," or "maybe" (uncertain eligibility). All records marked as "include" or "maybe" by either reviewer proceeded to full-text review. Disagreements were resolved through discussion, and a third reviewer (A.P.-P.) was consulted when consensus could not be reached.

Phase 3: Full-Text Review

The same two reviewers (B.F.-A. and D.N.-J) independently assessed the full text of all potentially eligible studies. Each study was evaluated against all inclusion and exclusion criteria, and reasons for exclusion were documented in Covidence. Disagreements were resolved through discussion, with arbitration by the third reviewer (A.P.-P.) when necessary.

2. Inter-Rater Reliability
Inter-rater agreement for study selection was assessed using Cohen's kappa (κ) statistic calculated in Covidence. Kappa values were calculated separately for title/abstract screening and full-text screening phases. Agreement was interpreted according to Landis and Koch criteria: $\kappa < 0.00$ (poor), 0.00–0.20 (slight), 0.21–0.40 (fair), 0.41–0.60 (moderate), 0.61–0.80 (substantial), and 0.81–1.00 (almost perfect). *Note: Final kappa values will be calculated upon completion of the screening process and reported in the Results section.*

3. Data Items
The following data items were systematically extracted from each included study:

Study characteristics:

- First author, publication year, country of origin
- Study design (in vitro, in vivo animal, clinical trial)
- Sample size (number of specimens or participants)
- Funding source and conflicts of interest

Orthodontic appliance characteristics:

- Type of appliance (bracket, archwire, mini-screw, aligner, etc.)
- Base material (stainless steel, nickel-titanium, ceramic, polymer, titanium alloy)
- Manufacturer and commercial name (if applicable)

Coating/intervention characteristics:

- Type of antibacterial agent (silver nanoparticles, TiO_2, ZnO, copper, chitosan, quaternary ammonium compounds, etc.)
- Coating method (physical vapor deposition, chemical vapor deposition, electrodeposition, sol-gel, dip coating, plasma treatment, etc.)
- Coating thickness and morphology (when reported)
- Concentration or loading of antibacterial agent
- Durability testing period (if assessed)

Bacterial strains and testing conditions:

- Bacterial species tested (*Streptococcus mutans*, *Lactobacillus acidophilus*, *Porphyromonas gingivalis*, *Aggregatibacter actinomycetemcomitans*, etc.)
- Culture medium and conditions (temperature, atmosphere, duration)
- Inoculum concentration

Outcome measures:

- Primary antibacterial outcomes: bacterial adhesion (CFU/cm^2), biofilm formation (optical density, crystal violet staining), zone of inhibition (mm), live/dead bacterial counts, SEM imaging scores
- Secondary outcomes: cytotoxicity, ion release, mechanical properties, surface roughness, clinical indices (plaque index, gingival index, white spot lesion scores)
- Quantitative results (mean ± standard deviation, median and interquartile range, or percentage reduction)
- Statistical significance (p-values, confidence intervals)
- Risk of bias domains (see Section 2.7)

When data were presented only in graphical form, values were extracted using WebPlotDigitizer software (version 4.6, Ankit Rohatgi, San Francisco, CA, USA). When critical data were missing or unclear, corresponding authors were contacted via email with a maximum of two reminder emails sent at two-week intervals.

Synthesis Approach

Due to substantial heterogeneity in study designs, orthodontic appliances, coating types, bacterial species, testing methods, and outcome measures, a narrative synthesis was deemed most appropriate. Meta-analysis was not feasible due to the diversity of interventions and outcomes, inconsistent reporting of quantitative data, and the pre-dominance of in vitro studies with non-standardized protocols. The narrative synthesis was conducted according to the Synthesis Without Meta-analysis (SWiM) reporting guideline [22]. Synthesis Structure is reported in the supplementary Materials.

Synthesis Structure

The synthesis was organized by:
- Type of orthodontic appliance: brackets, archwires, mini-screws, elastomeric ligatures, clear aligners, and removable appliances.
- Type of antibacterial coating: silver-based coatings, titanium dioxide (TiO_2), zinc oxide (ZnO), copper-based coatings, quaternary ammonium compounds, chi-tosan, graphene oxide, and combination coatings.
- Study design: in vitro studies, in vivo animal studies, and clinical trials.

Within each category, studies were grouped by coating type and bacterial species tested. For each group, we summarized:

-Range of antibacterial efficacy (percentage reduction in CFU, biofilm, or zone of inhibition - compared to controls)

-Consistency of findings across studies

-Durability of antibacterial effect over time

-Biocompatibility and safety concerns

-Methodological quality and risk of bias

Evidence Synthesis

As a scoping review, this study does not apply formal certainty-of-evidence frameworks such as GRADE (Grading of Recommendations Assessment, Development and Evaluation), which are designed for systematic reviews evaluating intervention efficacy and require homogeneous outcome measures amenable to meta-analysis [24]. Instead, evidence is synthesized narratively and presented in structured tables organized by:

- Orthodontic appliance type: Brackets, archwires, clear aligners, bands, mini-implants, elastomeric ligatures, removable appliances.
- Coating material category: Silver-based systems (nanoparticles, ions, composites), titanium dioxide-based systems (undoped, nitrogen-doped, metal-doped), zinc oxide-based systems, copper-based systems, polymer-based systems (chitosan, quaternary ammonium compounds, antimicrobial peptides), composite and hybrid systems.
- Study design: In vitro (further stratified by bacterial model and outcome measure), in vivo (animal models), clinical (human studies).

Within each category, the narrative synthesis reports:

- Range of antibacterial effects observed: For example, percentage reduction in colony-forming units (CFU), zone of inhibition (ZOI) diameter in millimeters, biofilm biomass reduction, or log reduction in viable bacteria. These ranges reflect the diversity of findings across heterogeneous protocols and should not be interpreted as pooled estimates or confidence intervals.
- Methodological diversity: Bacterial species tested (e.g., *Streptococcus mutans, Streptococcus sanguinis, Lactobacillus acidophilus, Porphyromonas gingivalis*, multi-species biofilms), assay types (CFU enumeration, ZOI, biofilm biomass, viability staining, microscopy, molecular assays), exposure durations (hours to months), and coating application methods (physical vapor deposition, electrochemical deposition, sol-gel, dip-coating, incorporation during manufacturing).
- Durability and stability findings: When reported, evidence regarding coating longevity under simulated or actual intraoral conditions, including thermocycling, pH cycling, mechanical abrasion, artificial saliva immersion, and intraoral exposure duration.
- Biocompatibility and safety findings: When reported, evidence from cytotoxicity assays (e.g., MTT, LDH release, live/dead staining with human cells), genotoxicity assays, animal studies, and clinical studies regarding adverse events, hypersensitivity reactions, or systemic absorption of coating components.

Synthesis prioritizes breadth over depth, consistent with the exploratory objectives of scoping reviews. The goal is to identify the full spectrum of approaches, highlight converging evidence where it exists, and transparently report divergent findings and knowledge gaps. No attempt is made to weight studies by quality or to calculate pooled effect sizes, as the heterogeneity of methods and outcomes precludes meaningful quantitative synthesis.

The narrative synthesis in the Results section (Section 3) is structured to facilitate comparison within appliance and coating categories, while the Discussion (Section 4) integrates findings across categories to identify overarching trends, mechanistic insights, and translational barriers.

Effect Direction Plots

To visualize the consistency and direction of effects across studies, effect direction plots were constructed for key comparisons (e.g., silver nanoparticle-coated brackets vs. uncoated brackets). Each study was represented by a symbol indicating the direction of effect (favoring intervention, favoring control, or no difference) and the risk of bias rating.

Subgroup and Sensitivity Analyses

Where sufficient data were available, subgroup analyses were planned to explore potential sources of heterogeneity:

-Coating method (physical vapor deposition vs. chemical methods vs. incorpo-ration into bulk material)

-Bacterial species (S. mutans vs. L. acidophilus vs. mixed-species biofilms)

-Study design (in vitro vs. in vivo vs. clinical)

-Risk of bias (low vs. moderate vs. high)

-Sensitivity analyses were planned to assess the robustness of findings by ex-cluding studies with high risk of bias or studies with critical methodological limitations.

Biological Relevance Tier Distribution

Application of the four-tier biological relevance hierarchy (Reported in the supplementary materials) to all 109 studies yielded the following distribution:

- Tier 1 (Proof-of-Concept): 42 studies: single-species planktonic, short-term (<24 h), no durability assessment, static immersion.
- Tier 2 (Mechanistic Evidence): 37 studies (34%): single-species biofilm (24–72 h), controlled conditions, no aging simulation.
- Tier 3 (Translational Evidence): 17 studies (16%): multi-species biofilm or salivary inoculum, durability assessed post-aging (thermocycling, mechanical abrasion), ≥2 weeks duration.
- Tier 4 (Clinical Validation): 13 studies (12%): in vivo animal models (n = 8) or human clinical trials (n = 5), ≥4 weeks duration, actual intraoral conditions.
