# Supplementary material for: Antibacterial Interventions for Orthodontic Appliances; Surface Modifications, Coatings and Bulk-Incorporated Antibacterial Agents: Materials, Mechanisms and Clinical Application—A Scoping Review
Source: Materials (Basel). 2026 Aug 27;19(17):3644. doi: 10.3390/ma19173644 (PMC13566774; doi:10.3390/ma19173644)
Supplement: Supplementary file 1 [file materials-19-03644-s001.zip › Supplementary File S3. Table S2 Complete Search Strategies.pdf]

Full Search Strategy

1. Overview

A comprehensive literature search was conducted across five electronic databases. The initial search was performed in December 2025, with a final update in May 2026. No date restrictions were applied to the search to maximize comprehensiveness. Language was restricted to English.

Database	Provider	Search Date	Records Retrieved
PubMed/MEDLINE	National Library of Medicine	December 2025; updated May 2026	—
Scopus	Elsevier	December 2025; updated May 2026	—
Web of Science Core Collection	Clarivate Analytics	December 2025; updated May 2026	—
Cochrane Central Register of Controlled Trials (CENTRAL)	Cochrane Library	December 2025; updated May 2026	—
Google Scholar	Google	December 2025; updated May 2026	First 200 results (ranked by relevance)
**TOTAL (before deduplication)**			**316**

All retrieved records were imported into **Covidence** (Veritas Health Innovation, Melbourne, Australia) for deduplication and screening management.

2. Search Strategy by Database

The search strategy combined three concept blocks using Boolean operators:

- **Block A:** Orthodontic appliances and devices
- **Block B:** Antibacterial agents, coatings, and surface modifications
- **Block C:** Antibacterial outcomes and endpoints

Both Medical Subject Headings (MeSH) terms and free-text keywords (Title/Abstract [TIAB]) were used to maximize sensitivity.

2.1. PubMed/MEDLINE — Full Search String

(

("Orthodontic Appliances"[MeSH] OR "orthodontic bracket*"[TIAB] OR "orthodontic archwire*"[TIAB] OR

"orthodontic wire*"[TIAB] OR "orthodontic band*"[TIAB] OR "orthodontic mini-implant*"[TIAB] OR

"orthodontic mini-screw*"[TIAB] OR "miniscrew*"[TIAB] OR "mini-screw*"[TIAB] OR

"clear aligner*"[TIAB] OR "Invisalign"[TIAB] OR "orthodontic aligner*"[TIAB] OR

"elastomeric ligature*"[TIAB] OR "orthodontic ligature*"[TIAB] OR

"orthodontic appliance*"[TIAB] OR "orthodontic device*"[TIAB] OR

"removable appliance*"[TIAB] OR "retainer*"[TIAB] OR "orthodontic retainer*"[TIAB] OR

"acrylic retainer*"[TIAB] OR "Hawley retainer*"[TIAB] OR

"nickel-titanium"[TIAB] OR "NiTi"[TIAB] OR "nitinol"[TIAB] OR

"stainless steel bracket*"[TIAB] OR "ceramic bracket*"[TIAB])

)

AND

(

("Anti-Bacterial Agents"[MeSH] OR "Nanoparticles"[MeSH] OR "Metal Nanoparticles"[MeSH] OR

"Silver"[MeSH] OR "Titanium"[MeSH] OR "Zinc Oxide"[MeSH] OR "Copper"[MeSH] OR

"Chitosan"[MeSH] OR "Surface Properties"[MeSH] OR "Coated Materials, Biomedical"[MeSH] OR

"antibacterial coating*"[TIAB] OR "antimicrobial coating*"[TIAB] OR

"antibacterial surface*"[TIAB] OR "antimicrobial surface*"[TIAB] OR

"surface modification*"[TIAB] OR "surface treatment*"[TIAB] OR

"silver nanoparticle*"[TIAB] OR "AgNP*"[TIAB] OR "nano-silver"[TIAB] OR "nanosilver"[TIAB] OR

"titanium dioxide"[TIAB] OR "TiO2"[TIAB] OR "TiO_2"[TIAB] OR "photocatalytic"[TIAB] OR

"nitrogen-doped TiO2"[TIAB] OR "N-doped TiO2"[TIAB] OR

"zinc oxide"[TIAB] OR "ZnO"[TIAB] OR "zinc oxide nanoparticle*"[TIAB] OR

"copper oxide"[TIAB] OR "CuO"[TIAB] OR "copper nanoparticle*"[TIAB] OR

"titanium nitride"[TIAB] OR "TiN"[TIAB] OR "TiN-Cu"[TIAB] OR

"graphene oxide"[TIAB] OR "GO coating*"[TIAB] OR

"chitosan coating*"[TIAB] OR "chitosan nanoparticle*"[TIAB] OR

"quaternary ammonium"[TIAB] OR "QAC"[TIAB] OR

"polyethylene glycol coating*"[TIAB] OR "PEG coating*"[TIAB] OR

"antimicrobial peptide*"[TIAB] OR "AMP coating*"[TIAB] OR

"ion implantation"[TIAB] OR "plasma immersion ion implantation"[TIAB] OR "PIII"[TIAB] OR

"physical vapor deposition"[TIAB] OR "PVD coating*"[TIAB] OR

"chemical vapor deposition"[TIAB] OR "CVD coating*"[TIAB] OR

"sol-gel coating*"[TIAB] OR "electrodeposition"[TIAB] OR "dip coating*"[TIAB] OR

"plasma treatment"[TIAB] OR "sputtering"[TIAB] OR

"gold nanoparticle*"[TIAB] OR "gold nanoclusters"[TIAB] OR

"curcumin nanoparticle*"[TIAB] OR "mesoporous silica"[TIAB] OR

"hydroxyapatite coating*"[TIAB] OR "HA coating*"[TIAB])

)

AND

(

("Biofilms"[MeSH] OR "Streptococcus mutans"[MeSH] OR "Bacterial Adhesion"[MeSH] OR

"Anti-Infective Agents"[MeSH] OR "Dental Caries"[MeSH] OR

"antibacterial activit*"[TIAB] OR "antimicrobial activit*"[TIAB] OR

"antibacterial effect*"[TIAB] OR "antimicrobial effect*"[TIAB] OR

"biofilm inhibition"[TIAB] OR "biofilm formation"[TIAB] OR "biofilm reduction"[TIAB] OR

"bacterial adhesion"[TIAB] OR "bacterial colonization"[TIAB] OR

"zone of inhibition"[TIAB] OR "colony forming unit*"[TIAB] OR "CFU"[TIAB] OR

"Streptococcus mutans"[TIAB] OR "S. mutans"[TIAB] OR

"Lactobacillus"[TIAB] OR "Porphyromonas gingivalis"[TIAB] OR

"white spot lesion*"[TIAB] OR "WSL"[TIAB] OR "enamel demineralization"[TIAB] OR

"plaque index"[TIAB] OR "gingival index"[TIAB])

)

Filters applied: English language; no date restriction.

2.2. Scopus — Search String

TITLE-ABS-KEY(

("orthodontic bracket*" OR "orthodontic archwire*" OR "orthodontic wire*" OR

"orthodontic mini-implant*" OR "orthodontic mini-screw*" OR "miniscrew*" OR

"clear aligner*" OR "elastomeric ligature*" OR "orthodontic appliance*" OR

"orthodontic retainer*" OR "NiTi" OR "nickel-titanium" OR "nitinol")

AND

("antibacterial coating*" OR "antimicrobial coating*" OR "antibacterial surface*" OR

"surface modification*" OR "silver nanoparticle*" OR "AgNP*" OR "nanosilver" OR

"titanium dioxide" OR "TiO2" OR "zinc oxide" OR "ZnO" OR "copper oxide" OR "CuO" OR

"titanium nitride" OR "TiN" OR "graphene oxide" OR "chitosan" OR

"quaternary ammonium" OR "ion implantation" OR "physical vapor deposition" OR

"sol-gel" OR "electrodeposition" OR "gold nanoparticle*" OR "curcumin nanoparticle*")

AND

("antibacterial activit*" OR "antimicrobial activit*" OR "biofilm inhibition" OR

"biofilm formation" OR "bacterial adhesion" OR "zone of inhibition" OR

"Streptococcus mutans" OR "white spot lesion*" OR "enamel demineralization" OR

"plaque index" OR "colony forming unit*")

)

AND LANGUAGE(English)

2.3. Web of Science Core Collection — Search String

TS=(

("orthodontic bracket*" OR "orthodontic archwire*" OR "orthodontic wire*" OR

"orthodontic mini-implant*" OR "orthodontic mini-screw*" OR "clear aligner*" OR

"elastomeric ligature*" OR "orthodontic appliance*" OR "orthodontic retainer*" OR

"NiTi" OR "nickel-titanium" OR "nitinol")

AND

("antibacterial coating*" OR "antimicrobial coating*" OR "surface modification*" OR

"silver nanoparticle*" OR "AgNP*" OR "nanosilver" OR "titanium dioxide" OR "TiO2" OR

"zinc oxide" OR "ZnO" OR "copper oxide" OR "titanium nitride" OR "TiN" OR

"graphene oxide" OR "chitosan" OR "ion implantation" OR "sol-gel" OR "electrodeposition")

AND

("antibacterial activit*" OR "antimicrobial activit*" OR "biofilm inhibition" OR

"bacterial adhesion" OR "zone of inhibition" OR "Streptococcus mutans" OR

"white spot lesion*" OR "colony forming unit*" OR "plaque index")

)

AND LA=(English)

2.4. Cochrane Central Register of Controlled Trials (CENTRAL) — Search String

#1 MeSH descriptor: [Orthodontic Appliances] explode all trees

#2 (orthodontic bracket* OR orthodontic archwire* OR orthodontic wire* OR clear aligner* OR

orthodontic mini-implant* OR orthodontic mini-screw* OR elastomeric ligature* OR

orthodontic retainer* OR NiTi OR nickel-titanium):ti,ab,kw

#3 #1 OR #2

#4 MeSH descriptor: [Anti-Bacterial Agents] explode all trees

#5 MeSH descriptor: [Nanoparticles] explode all trees

#6 (antibacterial coating* OR antimicrobial coating* OR silver nanoparticle* OR AgNP* OR

titanium dioxide OR TiO2 OR zinc oxide OR ZnO OR copper oxide OR titanium nitride OR

graphene oxide OR chitosan OR ion implantation OR surface modification*):ti,ab,kw

#7 #4 OR #5 OR #6

#8 MeSH descriptor: [Biofilms] explode all trees

#9 MeSH descriptor: [Streptococcus mutans] explode all trees

#10 (antibacterial activit* OR antimicrobial activit* OR biofilm inhibition OR bacterial adhesion OR

zone of inhibition OR Streptococcus mutans OR white spot lesion* OR colony forming unit*):ti,ab,kw

#11 #8 OR #9 OR #10

#12 #3 AND #7 AND #11

2.5. Google Scholar

The first 200 results ranked by relevance were screened for each of the following query strings:

1. "antibacterial coating" "orthodontic bracket"

2. "antimicrobial coating" "orthodontic archwire"
3. "silver nanoparticles" "orthodontic" "biofilm"
4. "titanium dioxide" "orthodontic" "antibacterial"
5. "zinc oxide" "orthodontic" "antibacterial"
6. "antibacterial" "clear aligner" OR "orthodontic aligner"
7. "antibacterial" "orthodontic mini-screw" OR "orthodontic mini-implant"
8. "antibacterial" "orthodontic retainer" OR "acrylic retainer"

3. Deduplication and Screening Flow

Stage	Records (n)
**Total records identified across all databases**	316
**Duplicate records removed (Covidence automated + manual)**	64
**Records removed by automation tools (ineligible)**	10
**Records removed for other reasons**	3
**Unique records screened (title/abstract)**	**239**
**Records excluded at title/abstract screening**	82
**— Not orthodontic appliances**	38
**— Not an antibacterial outcome**	29
**— Review articles**	15
**Reports not retrieved (full text unavailable)**	4
**Full-text articles assessed for eligibility**	**153**
**Full-text articles excluded**	44
**— Off-topic focus (dental materials without orthodontic application)**	18
**— Lack of primary experimental data (reviews, editorials, abstracts)**	12
**— No appropriate control group**	8

**— Non-English language**	4
**— Inaccessible full text despite author contact**	2
**Studies included in qualitative synthesis**	**109**
**— In vitro studies**	96
**— In vivo animal studies**	8
**— Clinical trials in humans**	5

4. Supplementary Search: Hand-Searching and Grey Literature

In addition to the electronic database searches, the following supplementary strategies were applied:

- **Reference list screening:** Reference lists of all included studies and of relevant narrative reviews identified during screening were hand-searched for potentially eligible studies not captured by the database search.
- **Citation tracking:** Forward citation tracking was performed for key included studies using Google Scholar and Scopus "Cited by" functions.
- **Journal hand-search:** The following journals were hand-searched for the period January 2024–May 2026 given their high relevance to the topic: *American Journal of Orthodontics and Dentofacial Orthopedics*, *European Journal of Orthodontics*, *Progress in Orthodontics*, *Angle Orthodontist*, *Journal of Orofacial Orthopedics*, *Dental Materials*, *Journal of Biomedical Materials Research*.
- **AI-assisted discovery:** Research Rabbit (web-based AI tool, 2024 version, Research Rabbit Inc.) was used for supplementary literature discovery based on semantic similarity to key included studies. All records identified through this tool were subjected to the same eligibility screening process as database records.

This supplementary search strategy is reported in accordance with PRISMA-ScR Item 8 and the SWiM reporting guideline.

5. Additional Search Methods

To ensure comprehensive coverage, the following supplementary search methods were employed:

1. **Citation searching:** Reference lists of all included studies and relevant review articles were manually screened to identify additional eligible studies not captured by database searches.
2. **Forward citation searching:** Google Scholar and Web of Science citation tracking were used to identify studies citing key included papers.
3. **Grey literature:** Conference abstracts from major orthodontic conferences (American Association of Orthodontists, European Orthodontic Society, World Federation of Orthodontists) from 2020–2025 were screened for unpublished data.
4. **Expert consultation:** Key authors in the field were contacted to identify ongoing or unpublished studies.
5. **Trial registries:** ClinicalTrials.gov and WHO International Clinical Trials Registry Platform (ICTRP) were searched for ongoing or completed clinical trials.

6.Study Selection Process

6.1. Screening Phases

Study selection was conducted in two phases using a systematic, standardized approach:

Phase 1: Title and Abstract Screening

- Two reviewers (BFA, APP) independently screened all titles and abstracts retrieved from database searches
- Studies were classified as: (1) Potentially eligible, (2) Clearly ineligible, or (3) Uncertain
- Studies classified as "potentially eligible" or "uncertain" by either reviewer advanced to full-text review
- Disagreements were resolved through discussion or consultation with a third reviewer (FJG)

Phase 2: Full-Text Review

- Two reviewers (BFA, APP) independently assessed full-text articles against predefined eligibility criteria

- Reasons for exclusion at full-text stage were documented using a standardized form
- Disagreements were resolved through discussion or third-reviewer arbitration (FJG)
- Inter-rater reliability was calculated using Cohen's kappa coefficient

Screening management: Covidence systematic review software (Veritas Health Innovation, Melbourne, Australia) was used to manage the screening process, remove duplicates, and track reviewer decisions.

6.2. Eligibility Criteria

Studies were included if they met the following criteria:

Inclusion criteria:

1. **Study design:** Experimental *in vitro* studies, animal (*in vivo*) studies, clinical trials (randomized controlled trials, non-randomized controlled trials, cohort studies, case-control studies), and pilot studies evaluating antibacterial coatings on orthodontic materials
2. **Population:** Any orthodontic material or appliance (brackets, wires, aligners, mini-screws, bands, ligatures, retainers)
3. **Intervention:** Application of antibacterial surface coating or surface modification technique
4. **Comparison:** Uncoated control or alternative coating
5. **Outcomes:** Quantitative assessment of at least one primary outcome (antibacterial efficacy, durability, or biocompatibility)
6. **Language:** Published in English
7. **Publication type:** Peer-reviewed journal articles, conference proceedings with full-text data

Exclusion criteria:

1. Studies evaluating antibacterial agents incorporated into bulk materials (e.g., mixed into adhesives or composite resins) rather than surface coatings
2. Studies focusing solely on surface characterization without antibacterial testing
3. Studies evaluating non-orthodontic dental materials (e.g., restorative materials, endodontic materials) without orthodontic application

4. Review articles, editorials, commentaries, letters to the editor, case reports, and opinion pieces (however, reference lists were screened for eligible studies)
5. Studies without quantitative outcome data (qualitative descriptions only)
6. Studies not published in English
7. Duplicate publications (most recent or most complete version retained)
8. Conference abstracts without available full-text data

6.3. Inter-Rater Reliability

Inter-rater agreement for study selection was assessed at both the title/abstract screening phase and the full-text review phase using Cohen's kappa (κ) coefficient. Kappa values were interpreted as: $\kappa < 0.20$ (poor agreement), 0.21–0.40 (fair), 0.41–0.60 (moderate), 0.61–0.80 (substantial), and 0.81–1.00 (almost perfect agreement).
