# Supplementary material for: Antibacterial Interventions for Orthodontic Appliances; Surface Modifications, Coatings and Bulk-Incorporated Antibacterial Agents: Materials, Mechanisms and Clinical Application—A Scoping Review
Source: Materials (Basel). 2026 Aug 27;19(17):3644. doi: 10.3390/ma19173644 (PMC13566774; doi:10.3390/ma19173644)
Supplement: Supplementary file 1 [file materials-19-03644-s001.zip › Supplementary File S4. Methodological characteristics extracted.pdf]

Methodological characteristics extracted for in vitro studies:

For each in vitro study, the following domains were systematically extracted and reported in the Results section (Tables 1–4):

Bacterial model complexity: Single-species reference strain (lowest translational relevance), single-species clinical isolate, defined multi-species biofilm, salivary-inoculated biofilm (highest translational relevance). The majority of included studies (n = 78/96; 81%) employed single-species reference strains of S. mutans or S. aureus, which provide proof-of-concept data but do not replicate the polymicrobial ecology of the orthodontic oral environment.

Biofilm maturation stage: Planktonic suspension (<24 h), early biofilm (24 h), mature biofilm (48–72 h), or extended biofilm (>72 h). Planktonic assays systematically overestimate antibacterial efficacy relative to biofilm models due to the absence of extracellular matrix protection.

Outcome measure type and quantification: Colony-forming unit (CFU) enumeration with stated detection limit, zone of inhibition (ZOI) diameter, biofilm biomass quantification (crystal violet, XTT), live/dead viability staining, scanning electron microscopy (SEM), or molecular assays (qPCR). These measures are not interchangeable; ZOI diameters and biofilm biomass values cannot be directly compared with CFU reductions.

Coating characterization completeness: Surface morphology (SEM/AFM), elemental composition (EDS/XPS), coating thickness, crystalline structure (XRD), adhesion strength, and ion release kinetics. Studies reporting ≥4 of these parameters were considered to have adequate physicochemical characterization.

Durability assessment: As-prepared coating only (no durability data), post-aging simulation (thermocycling, pH cycling, artificial saliva immersion), mechanical abrasion testing, or intraoral exposure. The majority of included studies (n = 64/96; 67%) tested coatings only in the as-prepared state without any durability challenge.

Statistical reporting: Sample size per group (n), number of independent replicates, statistical test employed, effect sizes, and confidence intervals. Studies reporting ≥3 independent replicates with inferential statistics were considered to have adequate statistical robustness.

Methodological characteristics extracted for in vivo and clinical studies:

For the 13 in vivo and clinical studies, the following additional domains were extracted:

Study design hierarchy: Randomized controlled trial (RCT) > non-randomized controlled trial > prospective cohort > retrospective cohort > case series.

Sample size and power calculation: Whether a formal sample size calculation was reported.

Follow-up duration: Short-term (≤4 weeks), medium-term (1–3 months), long-term (>3 months). No included study exceeded 12 weeks of follow-up.

Clinical endpoint type: Primary clinical endpoints (WSL incidence, caries increment, periodontal indices) versus surrogate endpoints (microbial counts). No included study used WSL incidence or caries increment as a primary endpoint.

Methodological safeguards: Blinding (participant, operator, outcome assessor), allocation concealment, intention-to-treat analysis, and loss-to-follow-up rate.

Evidence quality signal and summary observations:

The structured characterization revealed several patterns that inform the interpretation of reported efficacy values:

Studies using single-species planktonic assays reported systematically higher antibacterial efficacy (median CFU reduction: 85–99%) than studies using mature multi-species biofilms or salivary inoculum models (median CFU reduction: 40–70%), suggesting that published efficacy ranges overestimate real-world performance.

Only 33% of in vitro studies (n = 32/96) included any form of durability assessment beyond the as-prepared state.

Only 2 of 13 in vivo/clinical studies reported a formal power calculation.

No included clinical study used a primary clinical endpoint (WSL incidence, caries increment); all clinical studies relied on surrogate microbial outcomes.

These observations do not constitute a formal risk-of-bias assessment but provide a transparent, structured basis for contextualizing findings and identifying the most methodologically robust evidence within the reviewed literature. Readers are encouraged to weight findings from studies with higher bacterial model complexity, durability assessment, and clinical endpoint validity more heavily when interpreting the translational significance of reported results.
